# Structural, electrical, and magnetic properties of nano Sr_1−X_La_X_Fe_12_O_19_ (X = 0.2–0.8)

**DOI:** 10.1038/s41598-022-15250-2

**Published:** 2022-07-26

**Authors:** D. Baba Basha, N. Suresh Kumar, K. Chandra Babu Naidu, G. Ranjith Kumar

**Affiliations:** 1 College of Computer and Information Sciences, Majmaah University, Al’Majmaah, 11952 Kingdom of Saudi Arabia; 2 Department of Physics, JNTUA, Anantapuramu, A.P 515002 India; 3 Department of Physics, GITAM University, Bangalore Campus, Bangalore, 561203 India; 4 School of Applied Sciences, REVA University, Bangalore, 560065 India

**Keywords:** Nanoscale materials, Structural materials

## Abstract

The current work is mainly devoted to the synthesis, structural, electrical, and magnetic characterization of Sr_1−X_La_X_Fe_12_O_19_ (X = 0.2–0.8) (SLFO) nanoparticles synthesized via the hydrothermal technique. The hexagonal peaks were determined using X-ray diffraction analysis. The obtained results indicated that the lattice constants were noted to be increasing from 0.58801 to 0.58825 nm (a = b), and 2.30309 to 2.30341 nm (c) with increase of in ‘X’. The morphological studies ensured that the grains as well as nanoparticles of SLFO acquired almost spherical shape. The optical properties were investigated using FTIR and UV–Visible spectra. The optical bandgap (E_g_) of SLFO was found to be increasing from 1.866 to 2.118 eV with increase of dopant content. The electrical properties of SLFO were studied in detail as a function of temperature, and frequency. In addition, the dielectric modulus, and impedance spectroscopy analysis was carried out to describe the space charge polarization, and electric conduction mechanism, respectively. The hysteresis loop (M–H curves) of SLFO revealed the decrease of magnetization from 36.34 to 7.17 emu/g with increase in ‘X’.

## Introduction

Among all the magnetic materials, the hexaferrites are the special class of materials with high coercivity. Thus, these materials are popularly known as hard magnetic materials. Therefore, the hexaferrites got significant applications to manufacture permanent magnets. This kind of benefit was attributed to the parameters like high magnetization, magnetocrystalline anisotropy constant, inexpensive, thermal, and chemical stability^1^. Different properties such as crystal structure, particle size, surface morphology, preparation method, cation distribution etc., can reinforce to achieve the above-mentioned applications^1^. Generally, the hexagonal ferrites were categorized into six types viz., M (SrFe_12_O_19_), W (BaZn_2_Fe_16_O_27_), X (Ba_2_Mg_2_Fe_28_O_46_), Y (Ba_2_Co_2_Fe_12_O_22_), and Z (Ba_3_Mn_2_Fe_24_O_41_)^2^. The M-type hexaferrites come under hard ferrite category. The general chemical formula of M-type hexaferrite can be written as MFe_12_O_19_ (M = divalent elements like Ba, Sr, Pb, Zn, Mg, Ni etc.) which is like the magnetoplumbite structure^2^.

Due to prominent electrical, magnetic, optical, and electromagnetic properties, M-type hexaferrites got applications in microwave absorbers, filters, diagnostics, ferrofluids, transformer cores, magnetic memories, magnetic recording, and high frequency devices^3^. Few of these applications were obtained from the M-type hexaferrite compounds such as BaFe_12_O_19_, SrFe_12_O_19_, and PbFe_12_O_19_^4–23^. In addition, several scientists particularly focused on the synthesis and characterization of lanthanides (La, Sm, Gd, Nd, Pr etc.) doped SrFe_12_O_19_ (SFO) in order to enhance the hardness of the SFO^3^. As a result, few properties were improved significantly. However, the reports on La-doped SrFe_12_O_19_ nanoparticles related to electrical, optical, magnetic properties were not available in the literature in detail. Therefore, the authors focused on synthesis of SrLaFe_12_O_19_ nanoparticles for electrical, optical, magnetic, and impedance characterizations using the hydrothermal technique.

## Materials and experimental method

**Figure 1:**
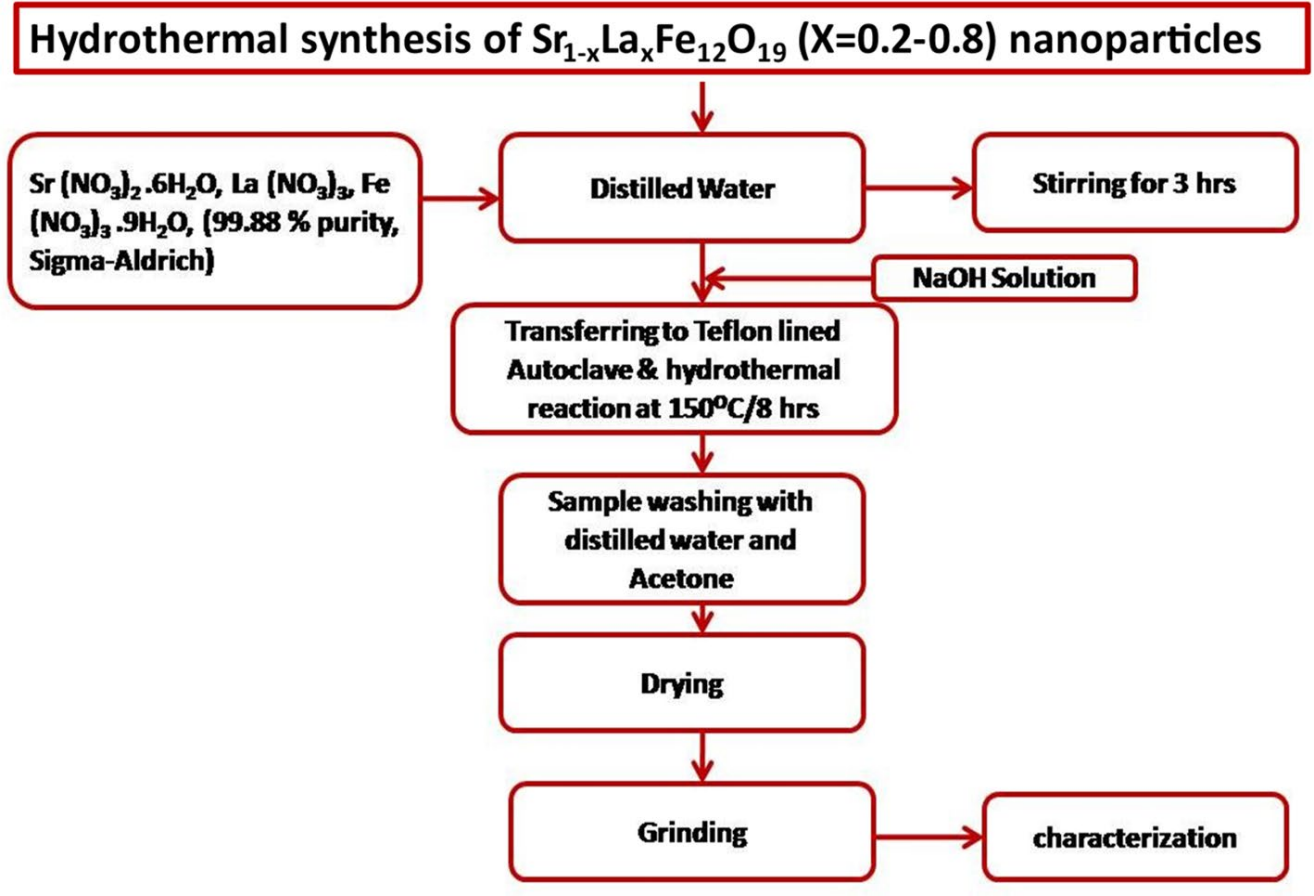
Schematic representation of synthesis of SLFO nanoparticles.

Hydrothermal synthesis technique is considered as one of the simplest and cost-effective technique to synthesize nanoparticles. The Sr_1−x_La_x_Fe_12_O_19_ (x = 0.2–0.8) (SLFO) nanoparticles were prepared using hydrothermal technique. In order to synthesize the SLFO nanoparticles, the precursor materials SrN_2_O_6_, LaN_3_O_9_, and FeN_3_O_9_ (each of 99.88% from Sigma-Aldrich) as mentioned in flow chart (Fig. 1) were chosen as per the stoichiometric-ratio. Different mass of nitrate materials was considered for x = 0.2–0.8 samples. Further, the precursors were taken into glass beaker containing 50 ml of deionized water. In order to mix the precursors, the glass beaker was kept on the magnetic stirrer. The solution was stirred for about 3 h. At the time of stirring, the NaOH-solution was added drop by drop to acquire stable pH-value (11). Afterwards, the obtained aqueous solution was kept in a Teflon bowl of 300 mL capacity which was enclosed in stainless-steel autoclave reactor. Later, the entire autoclave reactor was shifted into hot oven to perform hydrothermal reaction for 8 h. Throughout the reaction, the temperature of oven was maintained at 150 °C. After completion of the reacton, the oven was cooled down to room temperature naturally. Then, the obtained solution was cleaned several times by using distilled water and acetone to reduce the pH-value of the obtaied sample. This process was continued till pH reaches 7. In the next step, the sample mixed with limited water content was dried on the magnetic stirrer with hot plate by maintaing the temperature of 60 °C for two hours to remove the moisture present in the sample. The reason behind the heating is just to remove the remained water content. If not heated at low temperature, the moisture will certainly affect various electrical, optical, and magnetic properties. Further, the obtained sample was grinded to get fine powder. Finally, the nanoparticles in powder form were subjected to different characterization such as X-Ray diffraction (XRD) (Bruker, λ_CuKα_ = 0.15406 nm), TEM (Tecnai G20, FEI, USA), FESEM (Ultra 55, Carl Zeiss), FTIR (Shimadzu), UV–Visible spectrometer (JASCO, V-670 PC), LCR-controller (HIOKI 3532–50), and VSM (EV-7 H =  ± 15,000 Oe.) in order to disclose the phase, morphology, functional-groups, band gap, hysteresis-behavior, and electrical properties, respectively. The Fig. 1 illustrates the schematic representation of synthesis procedure of as prepared nanoparticles.

## Results and discussion

### X-ray diffraction (XRD) spectra

**Figure 2:**
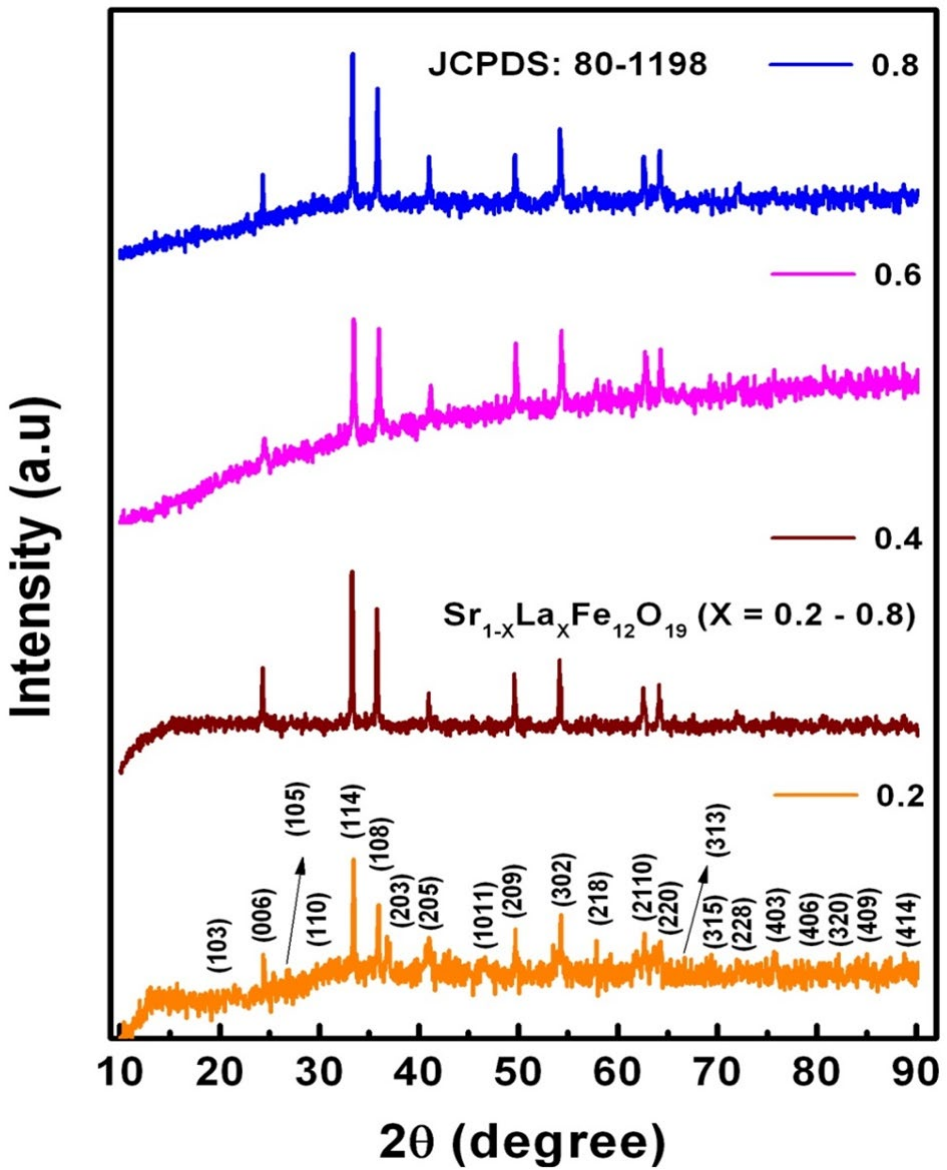
XRD spectra of SLFO nanoparticles.

**Figure 3:**
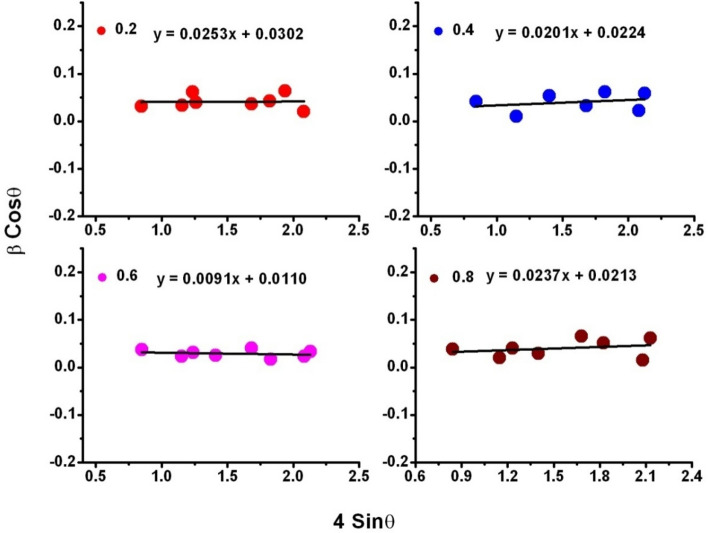
W–H plots of SLFO nanoparticles.

Figure 2 represents the XRD-spectra SLFO nanoparticles confirming the phase identification of prepared nanoparticles. These phases were indexed and compared with the standard JCPDS: 80–1198. From this, it was noted that all the diffraction phases were in consistent with the standard JCPDS. In addition, the highest counts were recorded for (114) reflection plane. The average crystallite diameter was calculated using Scherrer Eq., D_avg_ = 0.9λ_CuKα_/βcosθ, where λ_CuKα_ is the CuKα wavelength (0.15406 nm), β refers to full width half maxima (FWHM), and θ is associated to the diffraction angle^24^. The obtained D_avg_ values of x = 0.2–0.6 were found to be increasing from 4.5 to 14.8 nm. However, at x = 0.8, it was decreased to 5.2 nm. This can be usually attributed to the decreasing trend of microstrain (ε_s_) (from 0.0282 to 0.0210 rad) for x = 0.2–0.6 contents and increasing nature of the same to 0.0276 rad for x = 0.8 content. Likewise, the FWHM also followed the same trend as that of microstrain for all SLFO contents. This kind of behavior was earlier observed in the literature^25–29^. Besides, to provide a good agreement with these values, the microstrain (ε_W-H_), and average crystallite size (D_W-H_) parameters were calculated by plotting Williamson-Hall (W–H) plots as depicted in Fig. 3. These results were reported in Table 1. The achieved data suggested that both the D_W-H_ and ε_W-H_ values were in good consistent with the same parameters attained using the Scherrer method^24^.

**Table 1:** Data on structural parameters of SLFO nanoparticles.

X	0.2	0.4	0.6	0.8
D_avg_ (nm)	4.5	6.3	14.8	5.2
D_W-H_ (nm)	5.1	6.9	14.1	7.2
FWHM β_avg_ (°)	2.584	2.556	1.816	2.542
ε_s_ (radian)	0.0282	0.0270	0.0210	0.0276
ε_W-H_ (radian)	0.0253	0.0201	0.0091	0.0237
a = b (nm)	0.58801	0.58810	0.58818	0.58825
c (nm)	2.30309	2.30317	2.30328	2.30341
c/a ratio	3.9168	3.9163	3.9159	3.9157
MW (g/mol)	1072.016	1082.272	1092.504	1102.784
V_cell_ (nm)^3^	0.68960	0.68984	0.69006	0.69026
ρ_x_ (g/cm^3^)	5.162	5.210	5.257	5.305
ρ_b_ (g/cm^3^)	4.233	4.428	4.679	4.881
P (%)	18	15	11	8
P_avg_ (nm)	16.2	35.2	44.1	37.2
G_avg_ (nm)	23.5	109.3	117.6	102.4
S (m^2^/g)	258.3	182.9	77.1	217.5

Moreover, the lattice-parameters (a = b & c) were calculated using the equation: 1/d^2^ = [1.333/a^2^] [h^2^ + hk + k^2^ + (l^2^/c^2^)]^25^ and listed in Table 1. The obtained results indicated that the lattice-constants were noted to be increasing from 0.58801 to 0.58825 nm (a = b), and 2.30309 to 2.30341 nm (c) with increase in ‘X’. A detailed discussion can be given like this. Shannon ionic-radii table^30^ showed that the ionic-radii of cations of SLFO are noted as Sr^+2^ = 0.127 nm, La^+3^ = 0.122 nm, Fe^+3^ = 0.0645 nm, and Fe^+2^ = 0.080 nm. This data ensured that the La^+3^ ions have lesser ionic radii than Sr^+2^ ions and greater than the ionic radii of ferric and ferrous ions. Therefore, La^+3^ cations will have probability to occupy the Sr-site rather than the rest of the cationic sites. In the literature^31–35^, it was observed that the incorporation of rare earth cations into the site of divalent element can induce the conversion of Fe^+3^ ions to Fe^+2^ ions within the hexaferrite system. Subsequently, in the current study, it can be possible to replace the Sr^+2^ ions (high ionic radii) by La^+3^ ions (small ionic radii). However, one must understand a fact that the ionic radius of ferrous (Fe^+2^) ion is larger than ferric (Fe^+3^) ion. To form SLFO compound, it was clear that a greater number of Fe^+2^ ions should be formed^32^. Hence, the enhancement of lattice-constants was identified as a function of dopant composition. This kind of nature was noticed in the case of bulk SLFO material reported by Seifert et al.^31,32^. In some cases, the chemical composition, suppression effect of La^+3^ cations, and defects may also become accountable for present variation trend of unit-cell dimensions as well as its volume (V_cell_)^35^. The c/a ratio was found to be decreasing with increase in ‘X’. Furthermore, X-ray (ρ_x_ = ZM.W/N_A_V_cell_, where Z = effective-number of atoms per unit-cell, M.W = molecular-weight, N_A_ = Avogadro’s number, and V_cell_ = volume of the unit cell) and bulk densities (ρ_b_ is attained from Archimedes principle)^25^ were evaluated (see Table 1). The accomplished outcomes manifested that both the density parameters were noticed to be increasing with increase of La-content in SFO system. This was ascribed to the increase of molecular weight from 1072 to 1102.8 g/mol., as a function of ‘X’. In addition, the porosity (P = 1 −  (ρ_b_/ρ_x_)) was calculated and found to be decreasing from 18 to 8%. This confirmed a fact that the pore content was reduced upon increasing the La-content. Finally, the specific surface area (S) established the decreasing trend from ~ 258 to 77 m^2^/g (for X = 0.2–0.6). Beyond X = 0.6, the ‘S’ was about 217 m^2^/g. This nature was attributed to the increasing trend of crystallite size up to X = 0.6 and decreasing manner beyond X = 0.6. Similar reports were noticed in the literature^25^.

### Surface morphology

**Figure 4:**
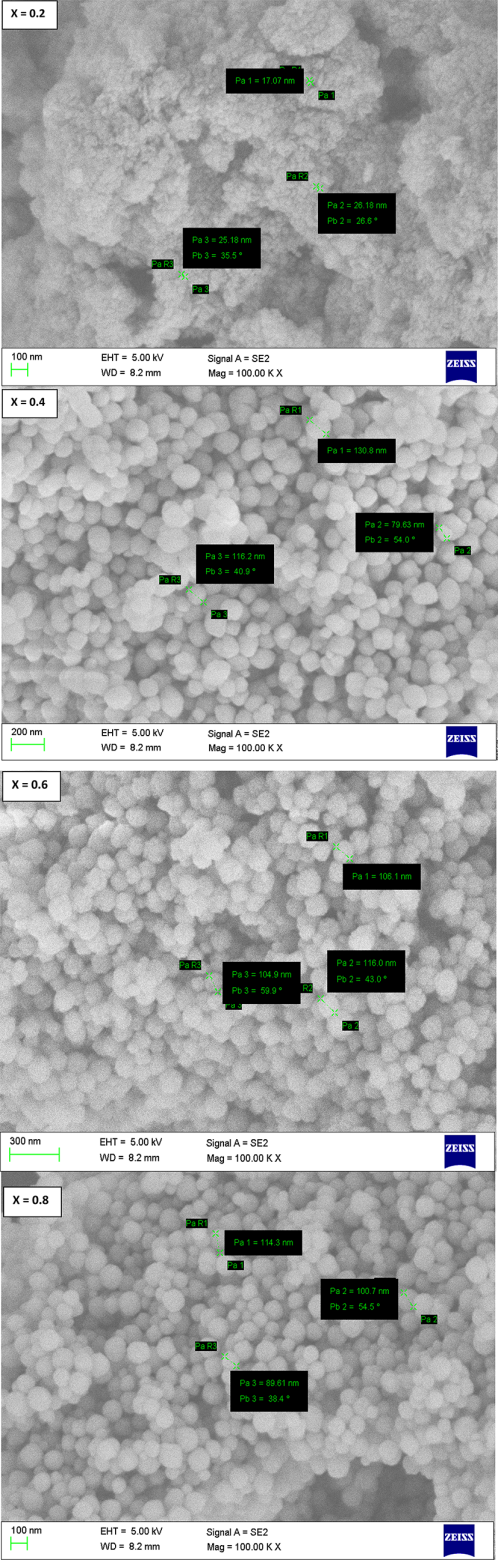
FESEM photos of SLFO nanoparticles.

Figure 4 showed the FESEM pictures of SLFO nanoparticles. In FESEM pictures, one can obviously notice that there were well-defined spherical shaped grains. Besides, these grains were distributed homogeneously. Comparatively, the X = 0.2 content acquired low apparent grain size while it seemed to be increasing from X = 0.2–0.6. But, for X = 0.8, it was decreased. Later, using linear intercept method, the average grain-size (G_avg_) was computed. In this technique, for each composition ten test-lines were drawn containing different test-lengths (L) at a specific working distance (WD) and magnification (M). Then, the total number of intersecting grains (N) was counted. Besides, all the parameters were inserted into the relation: G_avg_ = 1.5L/MN, where the symbols have their usual meaning. Thus, the experimental grain-size was accomplished and observed to be increased from 23.5 to 117.6 nm for X = 0.2–0.6. Furthermore, for X = 0.8, it was reduced to 102.4 nm. Similar kind of variation was noticed in average crystallite-size with increase in ‘X’ of SLFO-nanoparticles.

**Figure 5:**
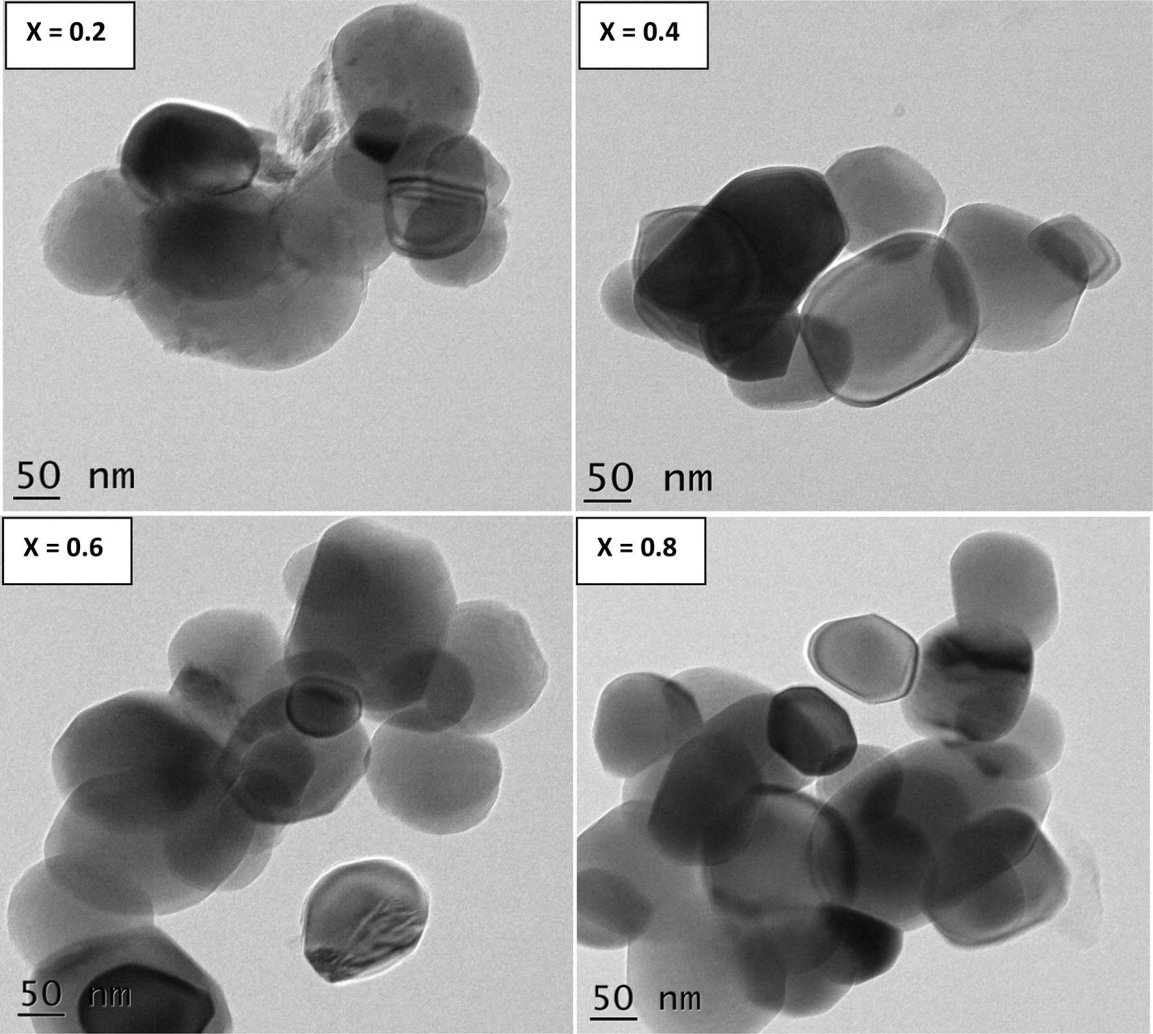
TEM photos of SLFO nanoparticles.

The TEM pictures as shown in Fig. 5 revealed the slightly distorted spheres like nanoparticles. Herein, the distribution of nanoparticles was seemed to be almost homogeneous. In addition, the average particle-size (P_avg_) was computed and noticed to be upsurging from 16.2 to 44.1 nm for X = 0.2–0.6 whereas the same was reduced to 37.2 nm for X = 0.8. This manner was identical to the variation of D_avg_ and G_avg_. Besides, the nanoparticles were appeared to be very close to each other. This can be normally attributed to various factors like magnetic interactions, agglomeration, size, charge etc.^25^. The samples are of same group of synthesized nanoparticles and processed with identical conditions. In general, the agglomeration of nanoparticles is responsible for little non-uniformity followed by distortions in the shape of nanoparticles shown in TEM pictures.

### Optical properties

**Figure 6:**
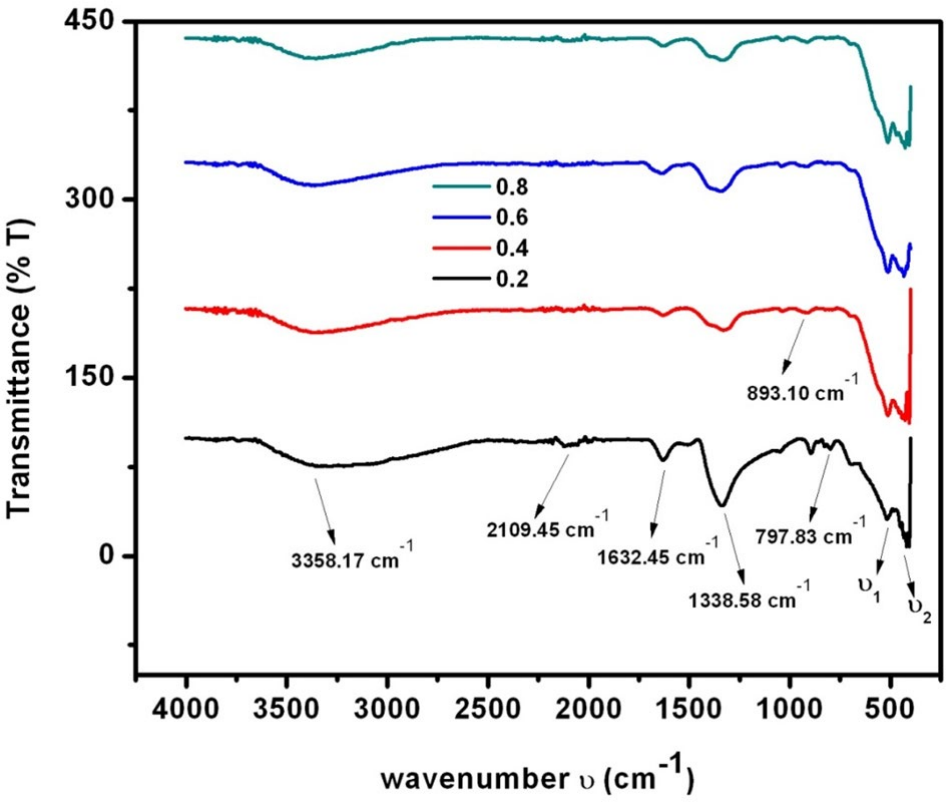
FTIR spectra of SLFO nanoparticles.

The Fourier-transform infrared-spectra (FTIR) of SLFO-nanoparticles were shown in Fig. 6. The FTIR-spectra evinced the formation of M-type hexagonal-structure. In which, the absorption bands located at 550 cm^−1^ were associated to Sr–O & La–O (M–O) site whereas, the bands located at 410 cm^−1^ were related to Fe–O site of hexagonal-structure (MFe_12_O_19_). Hence, for the prepared SLFO samples, the absorption bands noticed at ν_1_ were associated to the M-site while ‘ν_2_’ was associated to Fe–O bond. Therefore, this can indicate the development of hexagonal structure of prepared samples. Furthermore, as observed in Fig. 6, the formed additional bands on either side of iron-oxygen stretching vibrational site were ascribed to the presence of Fe^+2^-ions. Generally, this may be happened due to heat treatment of the samples. Also, few band sites were observed at 1632.5 & 2109.5 cm^−1^ and 3358.2 & 1338.6 cm^−1^. These sites were developed due to vibrational stretching which were appropriate to the contortion of water molecules and O–H bonds^25–27^.

**Figure 7:**
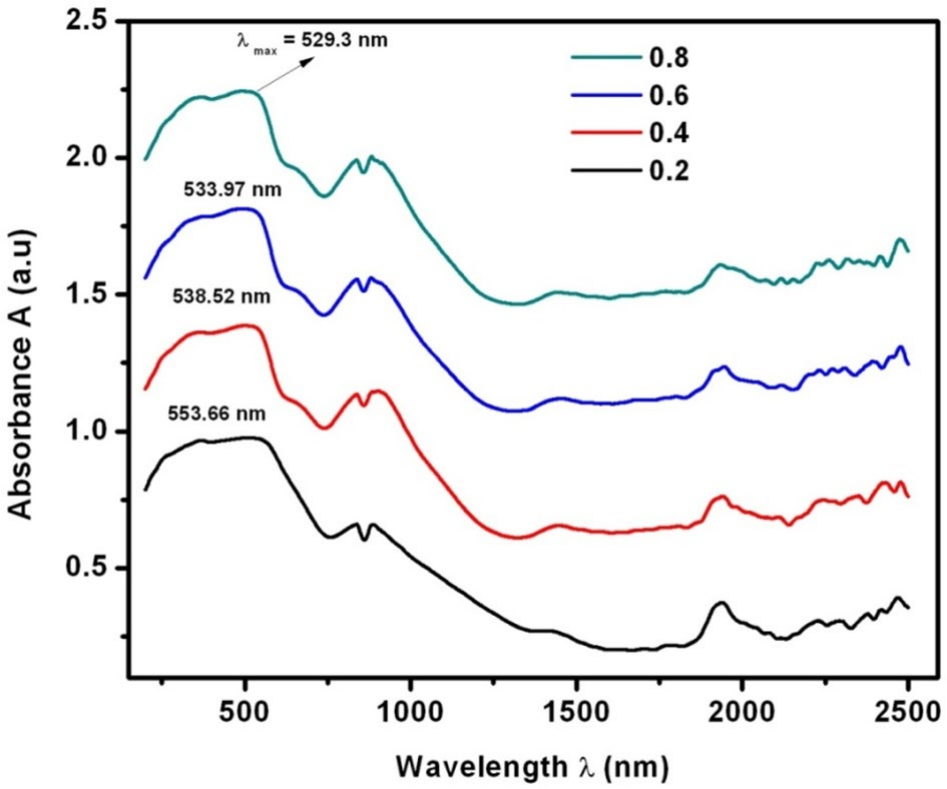
DRS spectra of SLFO nanoparticles.

**Figure 8:**
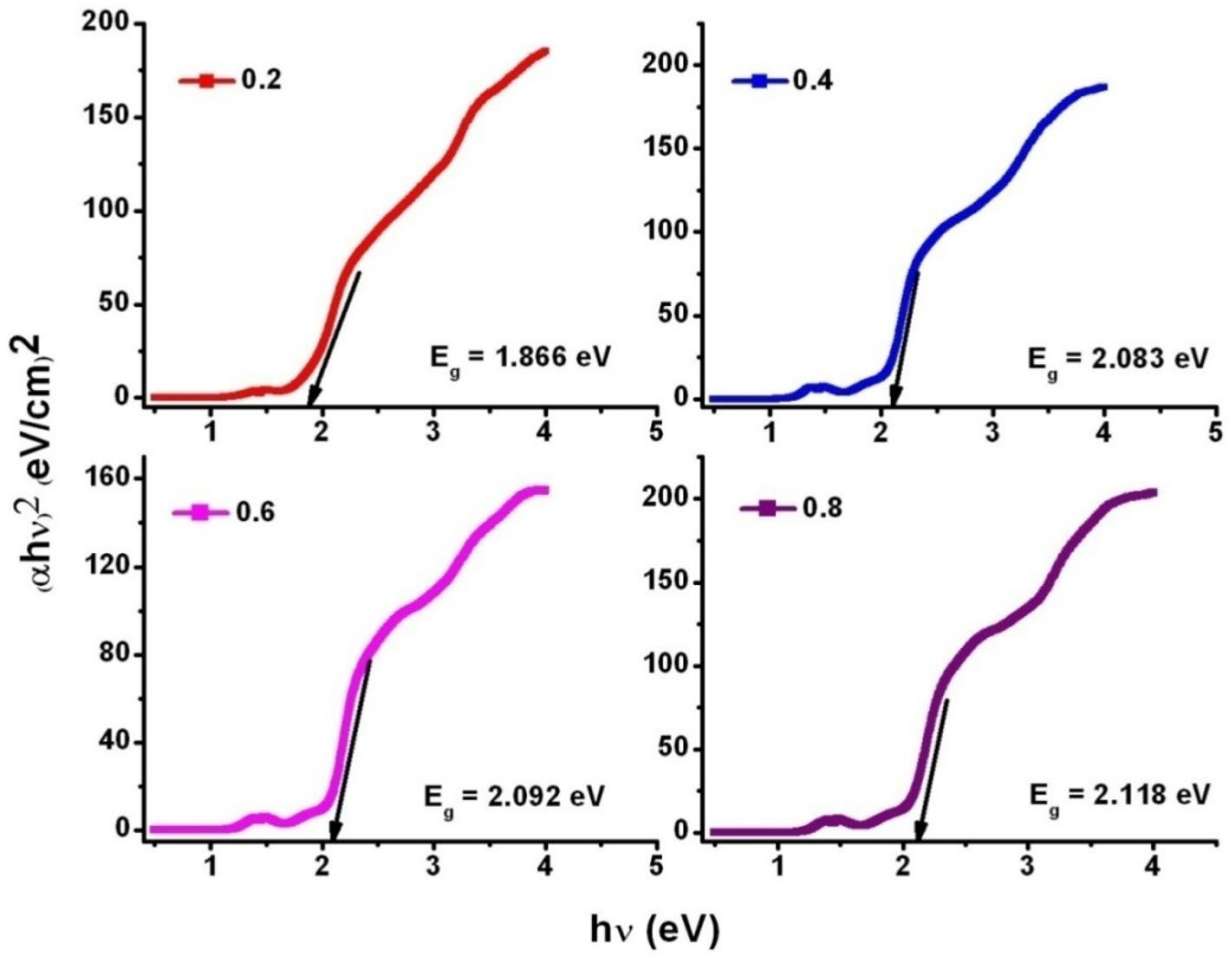
Optical bandgap determination of SLFO nanoparticles.

Figure 7 expressed the diffuse reflectance spectra (DRS) of SLFO nanoparticles. It was seen that the maximum absorption wavelength (λ_max_) was noted to be diminishing from 553.66 to 529.3 nm with increase of La-content. Furthermore, (αhυ)^n^ vs hυ plots (where ‘α’ is absorptivity, and ‘hυ’ is the photon energy) were drawn to obtain optical band gap energy (E_g_) of as prepared samples by considering n = 2. For n = 2, it allows the direct-transition of charge-carriers between the energy-bands^25–27^. Therefore, as shown in Fig. 8, we can mention (αhυ)^2^ versus hυ instead of (αhυ)^n^ versus hυ. From Fig. 8, it is clear the value of E_g_ was progressively increased from 1.866 to 2.118 eV with respect to dopant concentration. The value of E_g_ was obtained through extrapolation of linear portion of graph (Fig. 8) towards the axis of photon-energy, where the absorptivity tends to zero^26^. Therefore, the outcomes are evinced that the increasing La-content causes the enhancement in the value of E_g_.

### Magnetic behavior

**Figure 9:**
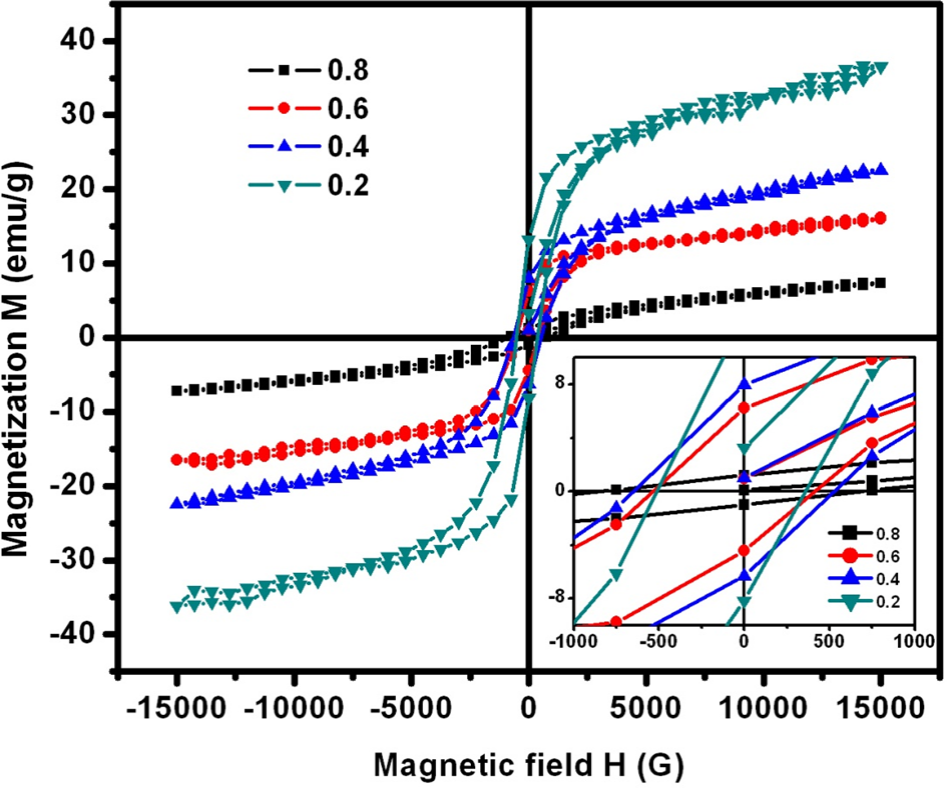
M-H loops of SLFO nanoparticles.

In case of SLFO nanoparticles, the magnetic nature was confirmed by hysteresis loops. These curves were plotted (see Fig. 9) using the magnetization (M) versus magnetic-field (H) data. In general, one can expect that the magnetization should be decreased upon increasing the nonmagnetic La-composition. From Fig. 9, it was seen that the saturation magnetization was decreasing from 36.34 to 7.17 e.m.u./g (see Table 2) with increase of La-content. Thus, the expectations became true in this case. As discussed in the “X-ray diffraction (XRD) spectra” section (XRD analysis), to replace the strontium site of large ionic radii by lanthanum of small ionic radii, there was an occurrence of conversion of ferric ions to ferrous ions. It was an established fact that Fe^+3^ ions contain the magnetic moment of 5μ_B_/f.u., while the Fe^+2^-ions attain magnetic-moment of 4 μ_B_/f.u. Indeed, this aspect implied a fact that the increasing La-content reduced the resultant magnetic moment thereby increasing the concentration of Fe^+2^ ions in SLFO. This manner was discussed earlier in the literature^31,32^ in case of bulk SLFO. Moreover, the increase of lattice constants with ‘X’ can be a second reason for the expansion of unit cell. Consequently, the magnetic exchange interactions will be decreased. This could be responsible for the reduction of magnetization thereby decreasing the magnetic moment of the spins. In addition, the high coercivity (H_c_) of SLFO was estimated to be changing from 490 to 820 G with dopant. This can be an indication for the hard ferrite nature of SLFO. Besides, the retentivity (M_r_) was found to be altering from 2.8 to 9.0 e.m.u./g. This suggested a fact that the variation of retentivity was unsystematic with increase in ‘X’.

**Table 2:** The magnetic and activation energy parameters of SLFO nanoparticles.

X	0.2	0.4	0.6	0.8
M_s_ (e.m.u./g)	36.34	23.02	16.14	7.17
M_r_ (e.m.u./g)	6.3	9.0	5.6	2.8
H_c_ (G)	820	540	650	490
E_a_ (eV) for H-region	0.154	0.189	0.190	0.147
E_a_ (eV) for L-region	0.0376	0.0432	0.0443	0.0274

### Temperature and frequency dependence of dielectric parameters

**Figure 10:**
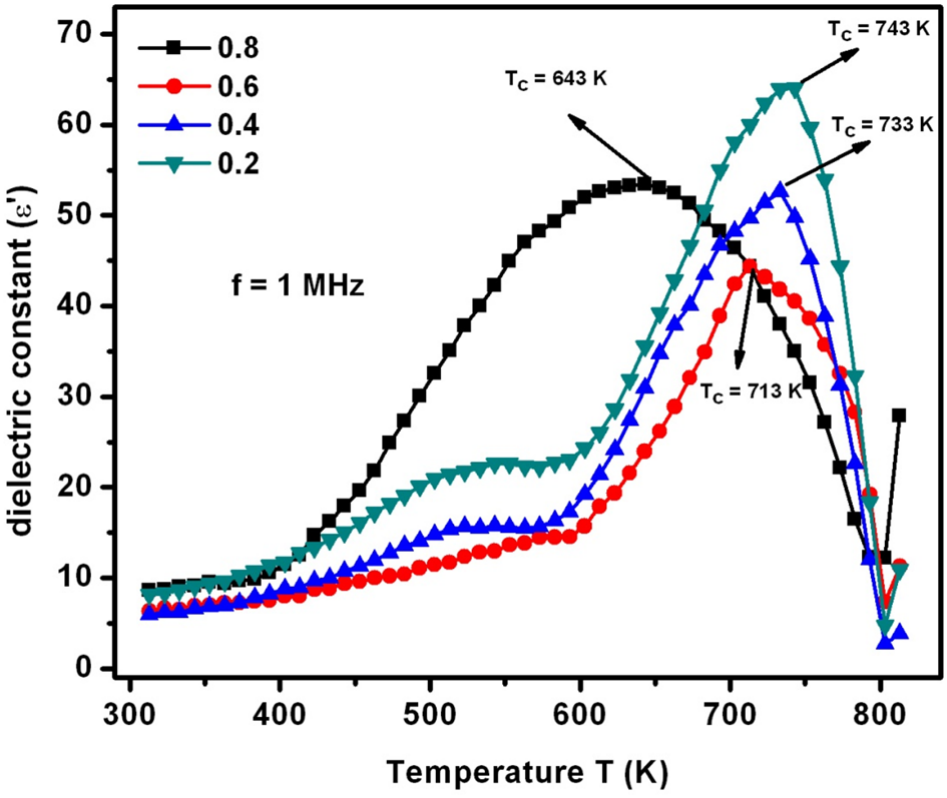
ε′−T plots of SLFO nanoparticles.

**Figure 11:**
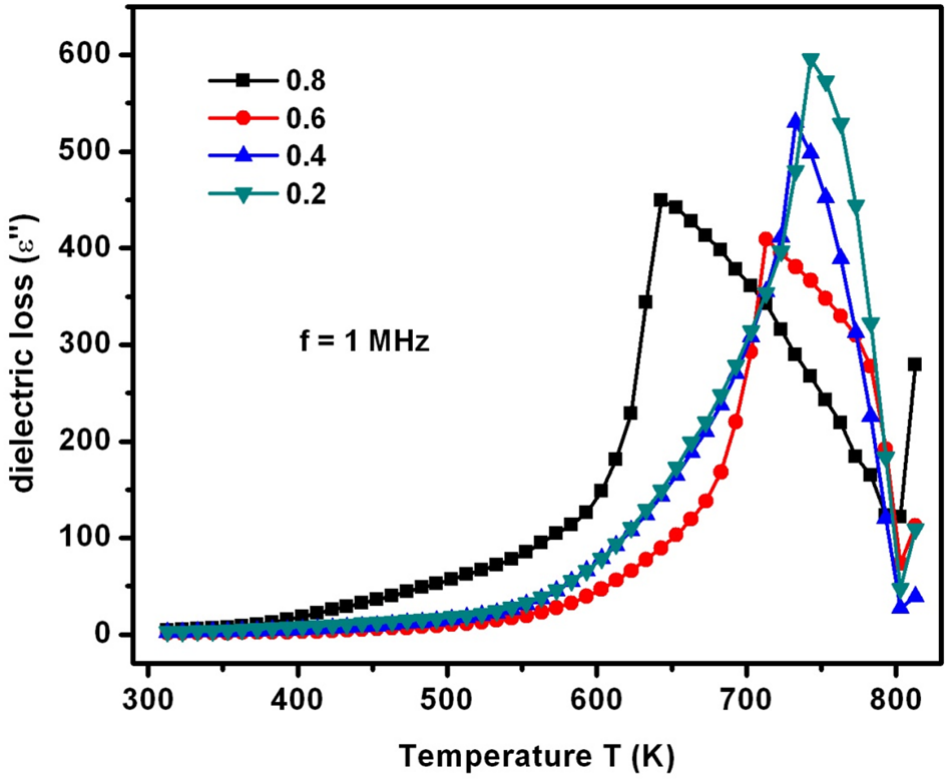
ε″−T plots of SLFO nanoparticles.

As a part of dielectric parameters, the dielectric constant (ε′), dielectric loss (ε″), ac-electrical conductivity (σ_ac_), and complex dielectric modulus (M*) were elucidated with respect to the variation of temperature (T) and frequency (f). It was a known fact that these parameters can mainly depend on distinct factors like sample preparation method, type of dopant, resultant compound, grain size, porosity, strain, density, and ionic radius^25^. Figure 10 depicted the ε′ versus T plots. It was observed from the ε′-T plots (at f = 1 MHz) that the ε′ of SLFO was remained constant until 400 K. It was attributed to weak and constant response of the charge carriers at these temperatures. Beyond 400 K, there was a gradual increasing trend of dielectric constant for X = 0.2–0.6. In contrast, X = 0.8 revealed an abrupt increasing manner of dielectric constant without any intermediate dielectric relaxations. Meanwhile, X = 0.2–0.6 contents exhibited dielectric relaxations between 533 and 583 K temperatures. These dielectric relaxations were established owing to high thermal agitations among the electric dipoles. As a result, the entropy becomes predominant at the relaxation temperatures which can in turn acquire the high magnitude of dielectric constant. Above 600 K, different compositions performed different Curie transition temperature (T_c_) values. That is, the T_c_ values were decreased from 743 to 643 K with increase of ‘X’ from 0.2 to 0.8. This kind of trend was obtained due to reduction in exchange-interactions between two sites of SLFO thereby increasing the lattice constants with increase of dopant concentration. Likewise, ε″ versus T plots (Fig. 11) stated almost similar T_c_ values as in case of dielectric constant versus temperature plots. Even, the variation trend of ε″−T plots was also identical to the ε′−T plots.

**Figure 12:**
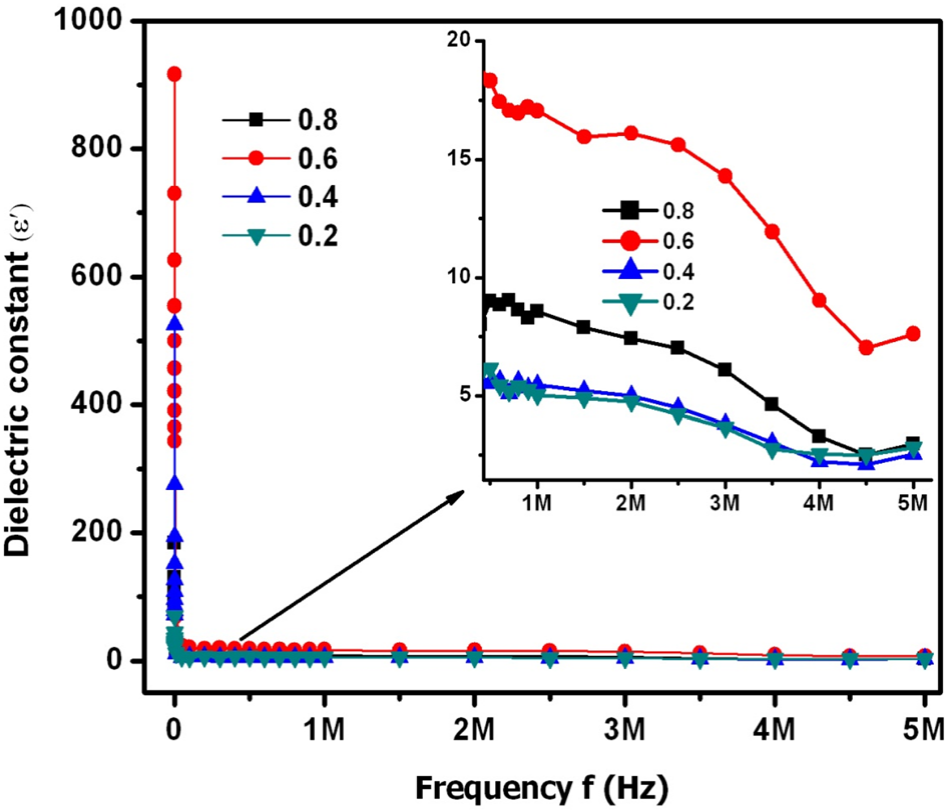
ε′−f plots of SLFO nanoparticles.

**Figure 13:**
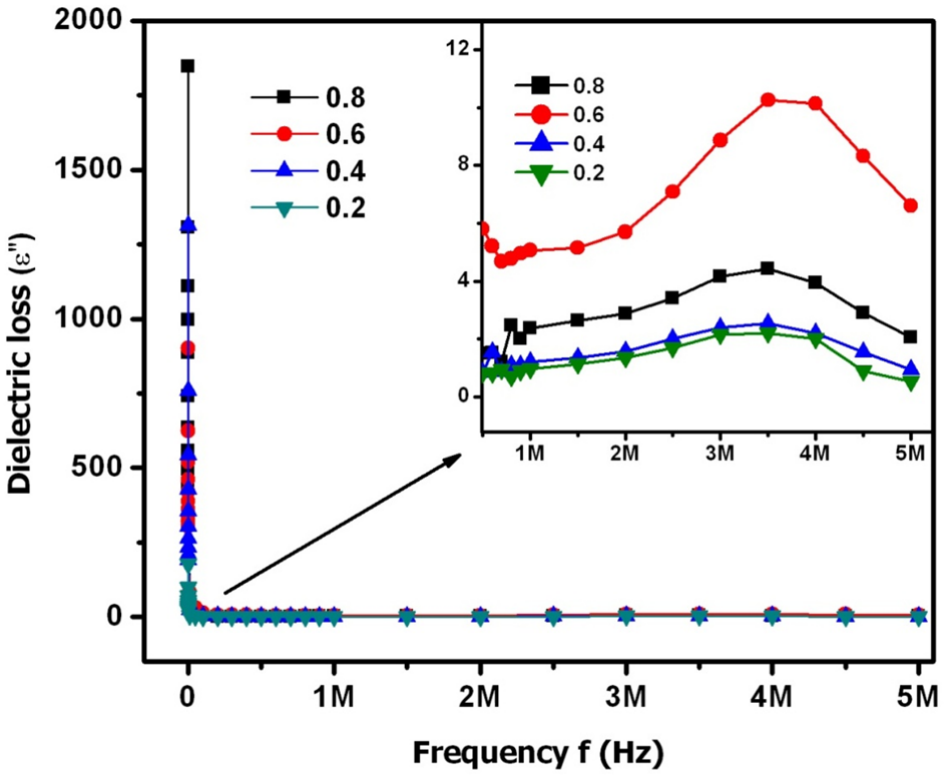
ε″−f plots of SLFO nanoparticles.

The frequency variation of dielectric constant and loss was described by means of ε′−f (Fig. 12) and ε″−f (Fig. 13) plots, respectively. In Fig. 12, it was noticed that the dielectric constant of X = 0.2–0.8 was decreasing from ~ 930 to ~ 30 at lower frequencies (between 100 Hz and 20 kHz). From this result, it was noticed that there was a maximum value of ε′ at 100 Hz while it was found to be 31 times smaller at 20 kHz. This kind of huge variation was earlier reported in Koop’s double-layer theory^36^. This theory reported that the polycrystalline material can be composed of two-layers such as (i) grain & (ii) grain-boundary. Indeed, these two layers are responsible for the higher and lower values of dielectric constant at lower and higher frequencies. The analysis of Koop’s theory suggested that the grain-boundaries are more resistive (low conductive) than the polycrystalline grains. Thus, at lower frequencies the charge carriers may not move and further they will be confined to certain microscopic region of the material. At this moment, the overcrowded nature of charge carriers usually takes place. This implied a fact that all the charge carriers were not able to break the barrier (grain boundary layer) due to its high resistance. Therefore, the grain boundaries were predominant at the low frequencies and consequently, it induced the huge amount of space charge or Maxwell–Wagner interfacial polarization. As a result, the maximum value of dielectric constant was possible at low frequencies. This type of discussion was earlier reported by Wagner^37^. On the other hand, with increase of input field frequency, the electric dipoles become more active after absorbing sufficient energy from the input field. Hence, the charge carriers can break the grain boundary layer which subsequently reduces the resistance of barrier. This kind of approach of carriers will induce to obtain the high conductivity. At this moment, the grains (referred as low resistive or conductive segments) become more active. Therefore, the space charge polarization will be diminished to larger extent. This in turn leads to achieve low dielectric constant value at high frequencies. After 20 kHz, the dielectric constant of all samples was seemed to be constant. However, the inset figure of Fig. 12 showed that X = 0.6 content revealed the high ε′ of ~ 18 at 1 MHz while the rest of the contents expressed moderate ε′ values varying from ~ 5 to 9. In Fig. 13, the ε″-f plots indicated that there was an observation of huge ε″ value altering from 1830 to 34 between 100 Hz and 20 kHz. On the other hand, above f = 20 kHz, almost a constant variation of loss was observed. This trend was just identical to ε′ behavior with ‘f’. However, the inset figure of Fig. 13 revealed a dielectric relaxation behavior at 3.5 for X = 0.2–0.8. Especially, X = 0.6 content performed the high loss of 10.28 at 3.5 MHz while X = 0.2, 0.4 & 0.8 contents showed the loss values from 1 to 4. The X = 0.6 content can be suitable for dielectric absorber applications at 1–5 MHz. Furthermore, this content may also work as microwave absorber at high frequencies.

**Figure 14:**
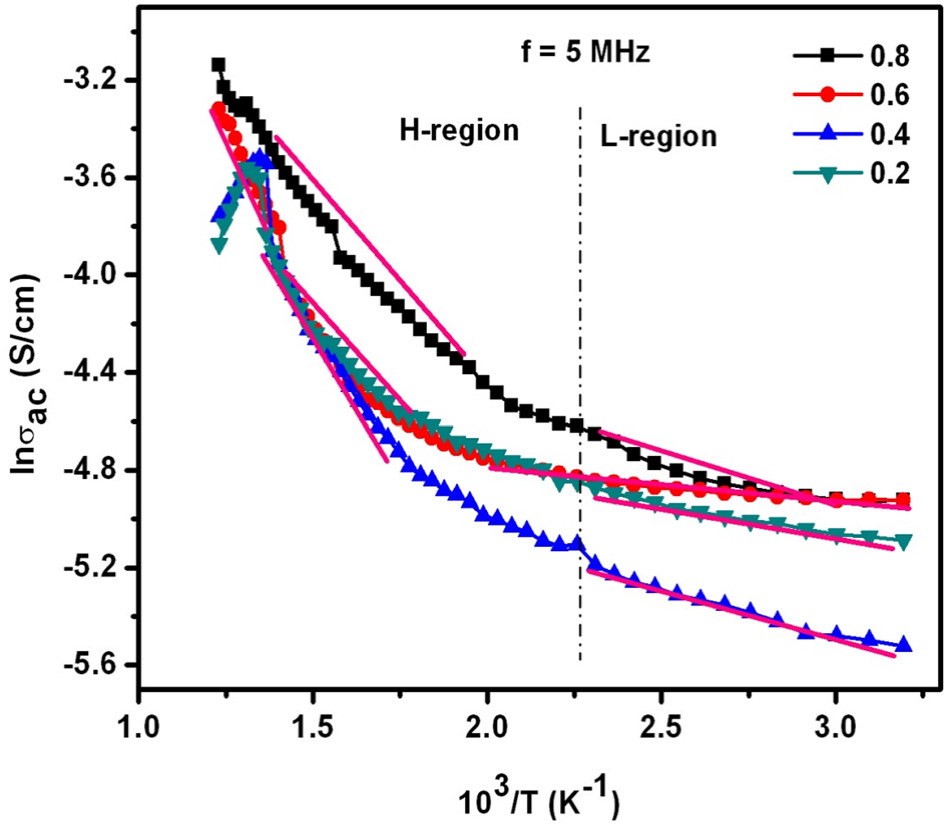
lnσ_ac_ versus 10^3^/T plots of SLFO nanoparticles.

Figure 14 indicated the behavior of σ_ac_ with temperature. It was clearly found that the conductivity was enhanced as a function of temperature. This was attributed to the thermal activation of charge carriers during the applied temperature range. Accordingly, hopping of electrons take place between ferric and ferrous ions. At very high temperatures, this electron hopping can be predominant and therefore, the conductivity goes to larger extent. Herein, the values of T_c_ were noticed to be decreasing from 743 to 643 K. Hence, one can expect the change of conduction mechanism before and after transition temperature. In view of this, the activation energies were determined by drawing Arrhenius-plots (see Fig. 14). In the plots, H-region corresponds to the high temperature while L-region is associated to the low temperature. In both the regions, the slopes were considered into an account. Furthermore, with the help of a standard equation E_a_ = 0.086 (slope), the activation energies (E_a_) were computed and depicted in Table 2. The obtained outcomes manifested that the values of E_a_ in H-region were detected to be increasing from 0.154 to 0.190 eV for X = 0.2–0.6 contents. But for X = 0.8 content, it was decreased to 0.147 eV. In the same way, the activation energies of L-region were also decreased from 0.0376 to 0.0443 eV for X = 0.2–0.6 compositions whereas, X = 0.8 exhibited E_a_ of ~ 0.0274 eV. From the results, one can understand that the E_a_ values of H-region seemed to be larger than that of L-region. This kind of low E_a_ values were obtained owing to the limited availability of charge carriers caused by magnetic disordering^25^. However, the L-region was evolved owing to the electrical conduction process produced by the extrinsic charge carriers while H-region was formed because of polaron hopping^25^. Previously, it was reported that there must be a change of slope of gradient line on passing through the T_c_^25^. In addition, the magnetic exchange interactions between the inner and outer electrons (e^−^) on either side of T_c_^25^ were responsible for the different activation energies. In other words, the change of magnetic state from ferri to para at the T_c_ can offer two different E_a_ values.

**Figure 15:**
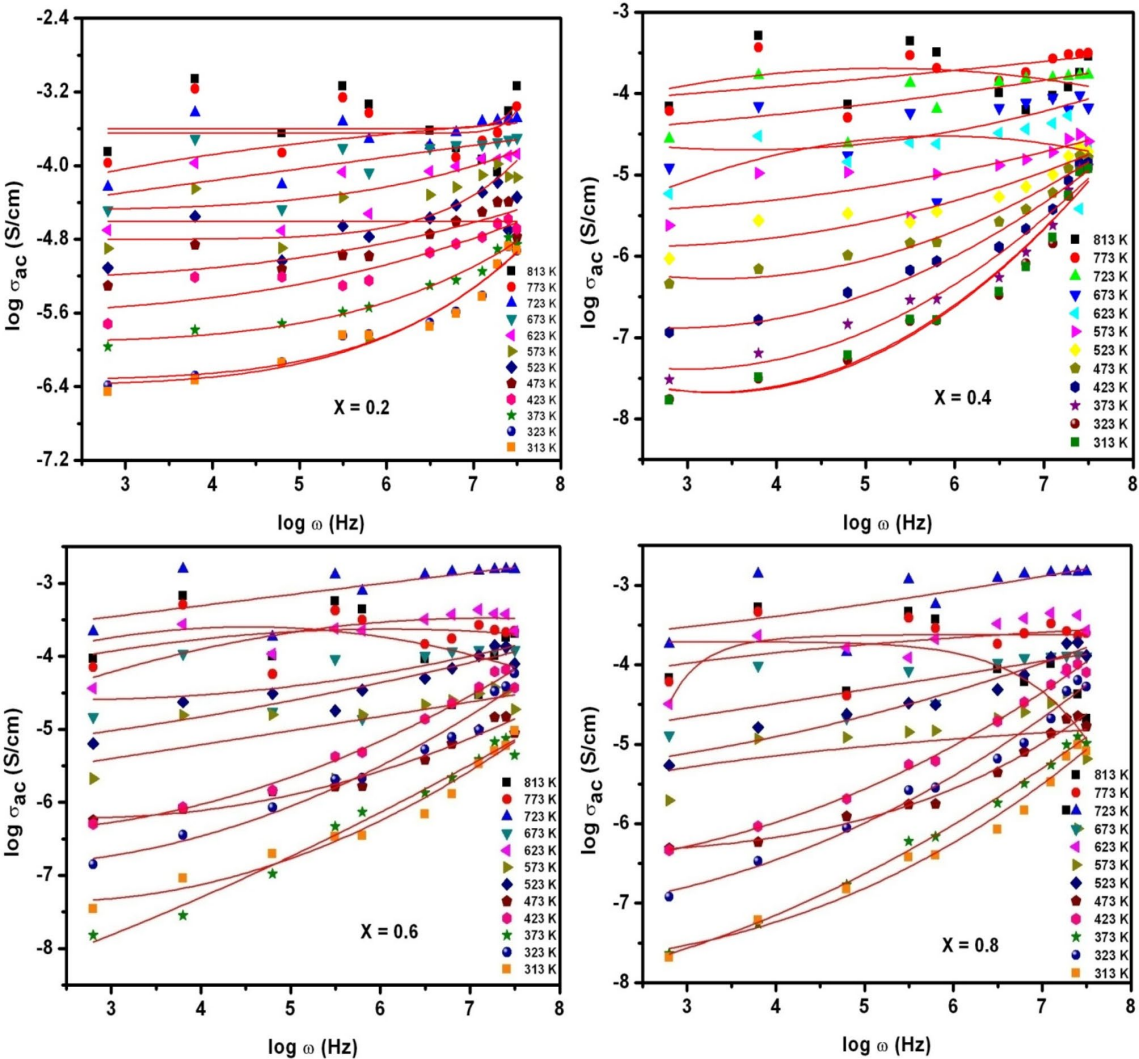
logσ_ac_ versus logω plots of SLFO nanoparticles.

As depicted in Fig. 15, the power-law fit was accomplished to log σ_ac_ − logω plots of X = 0.2–0.8 at various temperatures ranging from 313 to 813 K. Actually, the graphs from Fig. 15 can provide dc-conductivity (σ_dc_) and exponent (n) values. It was well-known fact that σ_ac_ is the combination of σ_dc_ (T) (temperature-dependent) & σ_ac_ (f) (frequency-dependent) terms. Mathematically, it can be written as follows: σ_ac_ (f, T) = σ_dc_(T) + σ_ac_(f)^25^. For all the temperatures, the frequency-independent term in Fig. 15 can be identified from the invariant portion of the log σ_ac_–logω plots. The computed σ_dc_ was found to be increasing from lower values to higher values as depicted in Table 4. That is, for X = 0.2–0.8, the σ_dc_ was noticed to be almost increasing from 4.18E−07–2.54E−04, 1.72E−08–7.19E−05, 2.75E−08–6.51E−05 and 1.59E−08–1.95E−04 S/cm, respectively. This established a fact that low σ_dc_ was recorded for x = 0.6 & 0.8 (large La-content). Moreover, exponent (n) values were also calculated and reported in Table 3. It was clear from the obtained ‘n’ values that it was decreasing from high value to low value for all La-contents. This was in consistent with the reports made by Hiti^38^. As a whole, the ‘n’ value offers the ratio between back hop-rate and site-relaxation. Therefore, it can achieve a maximum value of ‘1’ & minimum value of ‘0’. In current work, the value of ‘n’ was accomplished to be less than one for all ‘T’ values (313–813 K). This established a fact that site-relaxation happened in SLFO-nanoparticles was quicker than the hopping of polarons^25^.

**Table 3:** DC-electrical conductivity and exponent parameters of SLFO nanoparticles.

T (K)	0.2		0.4		0.6		0.8	
	σ_dc_ (S/cm)	n	σ_dc_ (S/cm)	n	σ_dc_ (S/cm)	n	σ_dc_ (S/cm)	n
813	2.54E−04	0.415	7.19E−05	0.342	6.51E−05	0.368	1.95E−04	0.280
773	2.25E−04	0.434	5.86E−05	0.465	3.13E−05	0.386	5.62E−05	0.456
723	3.71E−05	0.462	2.72E−05	0.413	1.18E−04	0.458	1.67E−04	0.581
673	3.69E−05	0.578	1.94E−05	0.429	2.68E−05	0.653	7.05E−07	0.553
623	2.62E−05	0.616	1.40E−07	0.498	2.98E−05	0.609	2.41E−04	0.550
573	1.59E−05	0.645	4.12E−07	0.552	3.03E−06	0.679	4.19E−06	0.659
523	1.57E−05	0.639	1.49E−06	0.510	3.46E−06	0.735	3.87E−07	0.687
473	5.87E−06	0.689	4.35E−07	0.548	4.16E−07	0.720	4.25E−07	0.669
423	2.49E−06	0.779	1.22E−07	0.626	3.89E−07	0.789	2.44E−07	0.764
373	1.21E−07	0.716	3.91E−08	0.790	8.36E−09	0.852	1.75E−08	0.866
323	4.77E−07	0.780	2.39E−08	0.751	1.04E−07	0.823	6.68E−08	0.829
313	4.18E−07	0.852	1.72E−08	0.746	2.75E−08	0.841	1.59E−08	0.873

### Dielectric and impedance spectroscopy analysis

**Figure 16:**
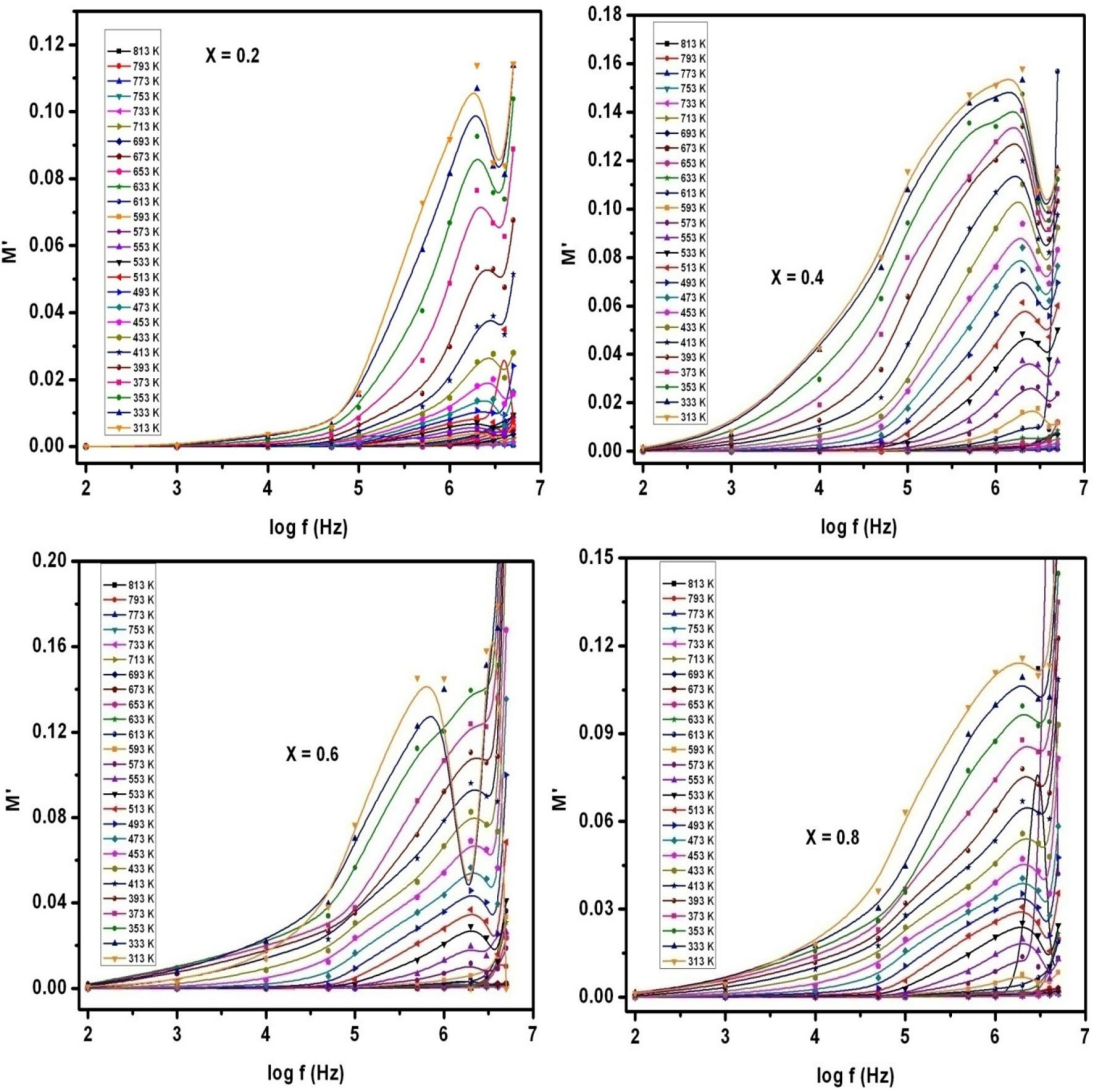
M′ versus log f plots of SLFO nanoparticles.

**Figure 17:**
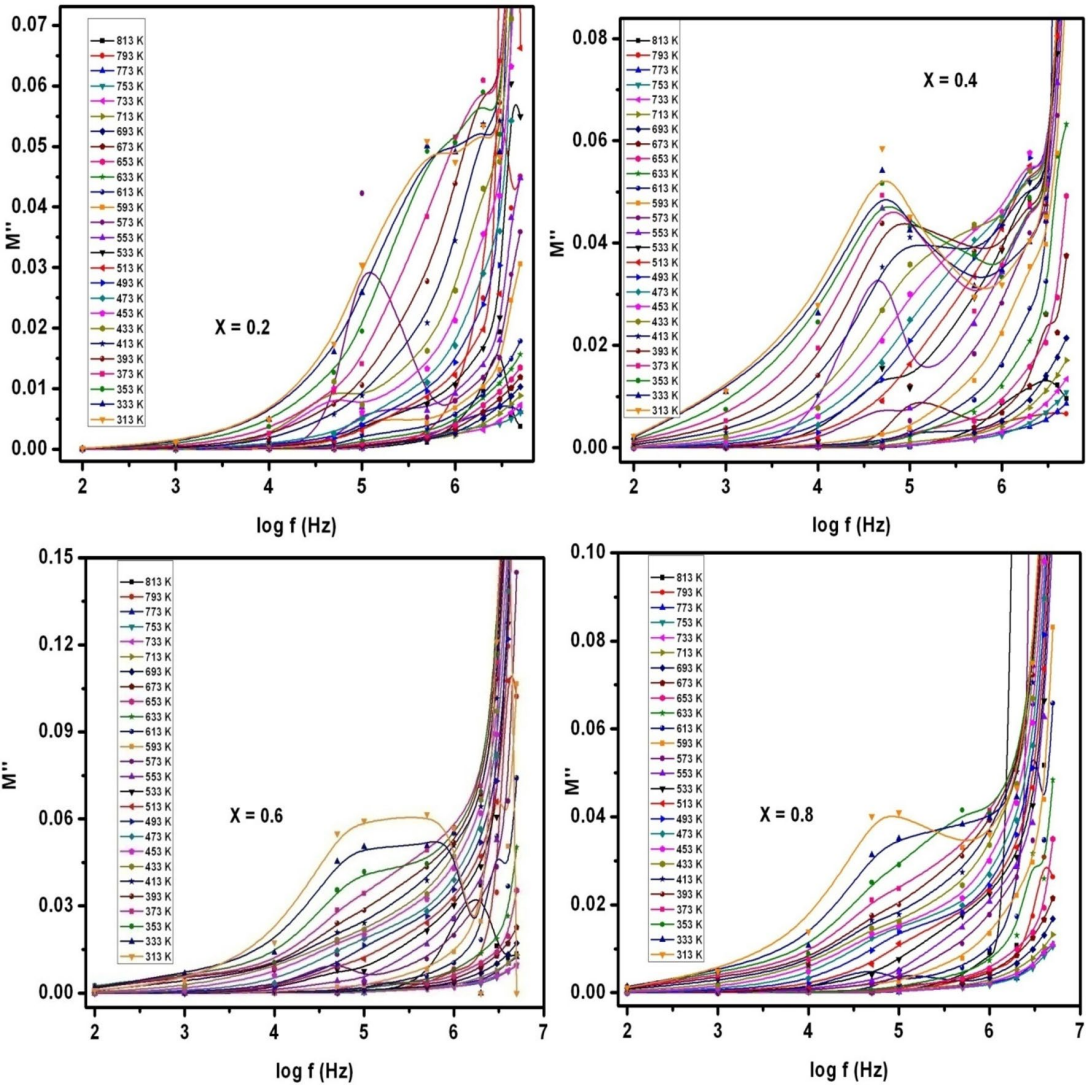
M″ versus log f plots of SLFO nanoparticles.

The complex dielectric modulus (M^*^) is normally written as M* = M′ + j M″, where M′ = (ε′/(ε′^2^ + ε″^2^)) and M″ = (ε″/(ε′^2^ + ε″^2^)). The electrical conduction as well as the space charge polarization effect can be well understood by studying the complex dielectric modulus formalism. The real and imaginary parts of dielectric modulus (M′ & M″, respectively) were deliberated in case of SLFO nanoparticles. The related graphs were depicted in Figs. 16 and 17. Figure 16 indicated that the real part of dielectric modulus versus input field frequency plots (M′−f) of X = 0.2–0.8 showed relaxation (resonance) behavior. Therefore, the complete plots were divided into couple of regions and the corresponding frequency was considered as relaxation frequency (f_r_). These two regions were denoted as region-a (< f_r_) and region-b (> f_r_). The M′−f plots of X = 0.2–0.8 contents established a fact that the resonance frequencies were increased towards higher frequencies as a function of temperature from 313 to 813 K. It was practically seen that in case of X = 0.2–0.8, the f_r_ values were (≥ log f) observed as 6.27, 6.16, 5.81, and 6.22, respectively. These f_r_ values were noted to be decreased from X = 0.2–0.6 and beyond that it was increased to log f = 6.22 at room temperature. Usually, it was an established fact that the relaxation frequencies can be identified owing to the charge carrier accumulation at the grain-boundary interface. Thus, the space-charge polarization becomes predominant, and it can further show huge value of M′. In the same way, these kinds of relaxation were recorded in M″−f plots (see Fig. 17) up to smaller extent. That means, the significant relaxations were seen at low temperatures (see X = 0.4 content) while small relaxations were observed at large temperatures. However, the space charge polarization mechanism was found in these materials. Herein, the low frequency relaxations specified a fact that the space-charges were triggered for small input field frequency of log f = 5 and further accumulated at the interface. Further, it was found that the small M′-values were recorded at low f-values (< 1 kHz). This was attributed to the electrode polarization effect. Moreover, the regions below log f = 6.27, 6.16, 5.81, and 6.22 (see M′-f plots) can be dedicated to the region of long-range polarization. Inside this region, one can recognize the long-range hopping conduction mechanism which was grown due to the long-range mobility of charge-carriers. Likewise, M″−f plots disclosed the small relaxations owing to long-distance motion of ions. Conversely, high f_r_-values were determined in case of M′−f & M″−f plots. These were formed owing to presence of ions confined to potential well. This approach was found in the previous reports^25^. Moreover, the region beyond f_r_ was noted as short-range polarization region where the short-range mobility of charge-carriers can be originated. Also, this can reflect the short-range hopping conduction mechanism.

**Figure 18:**
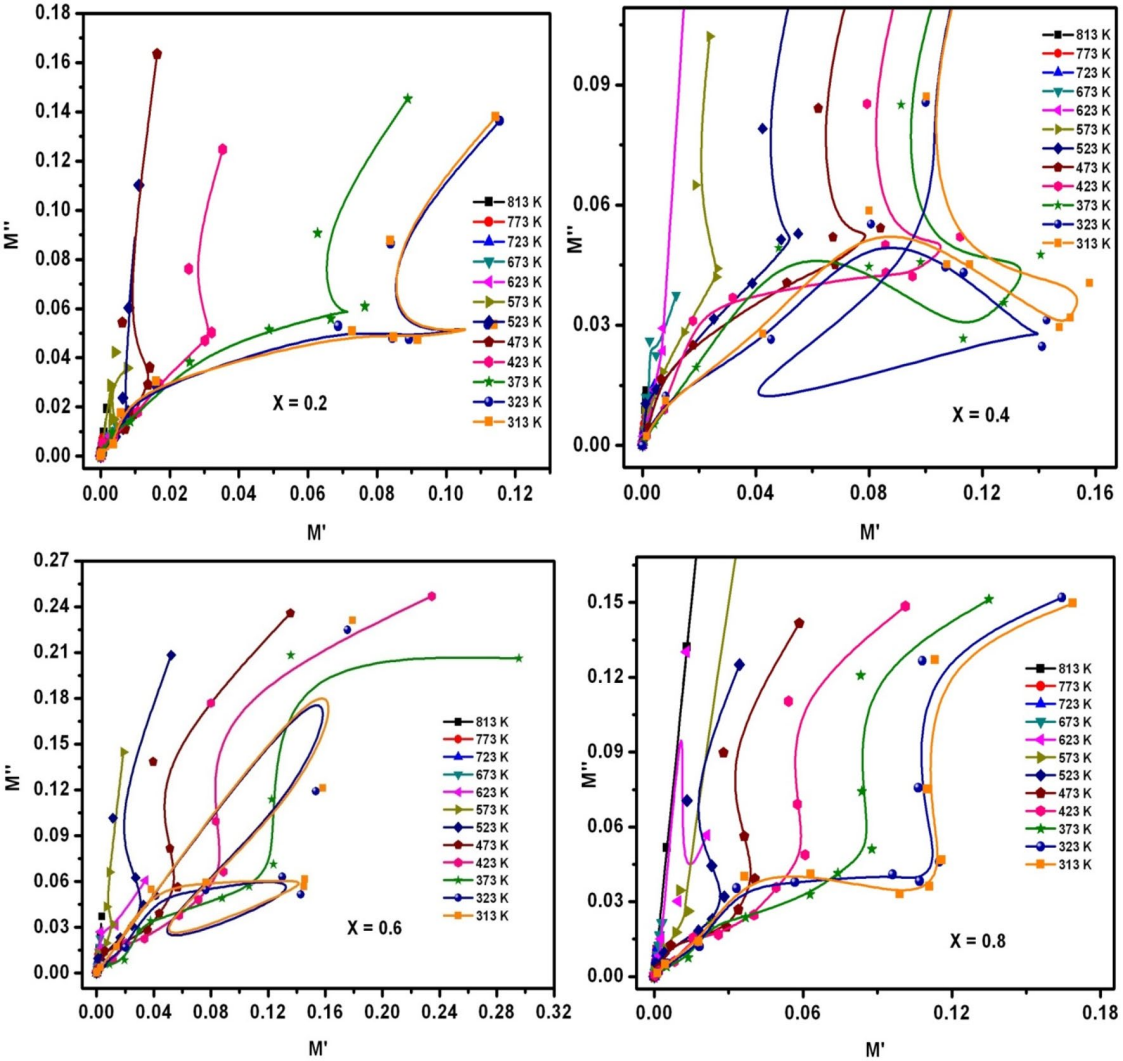
M′ versus M″ versus log f plots of SLFO nanoparticles.

In order to discuss the behavior of electrical conduction and relaxation, the M′ versus M″ plots were drawn as shown in Fig. 18. In Fig. 18, the semicircular arcs were associated to the contribution of grain & grain-boundaries in the electrical conduction-mechanism. Nevertheless, the noticed relaxations were partial in nature. Usually, these were developed due to partial relaxation-strength. Further, the presence of non-Debye relaxations in the prepared samples were confirmed from Fig. 18 i.e., the centers of semicircles were existed below the M′-axis. Specifically, the first semicircular-arc was connected to induced electrical-conduction owing to grain-contribution whereas the second one was related to the contribution of grain-boundary in the conduction mechanism. Also, some distortions were noticed in M′ versus M″ plots, which were evolved owing to intrinsic factor such as micro-strain, pores, temperature, grain-size and moisture^25^.

**Figure 19:**
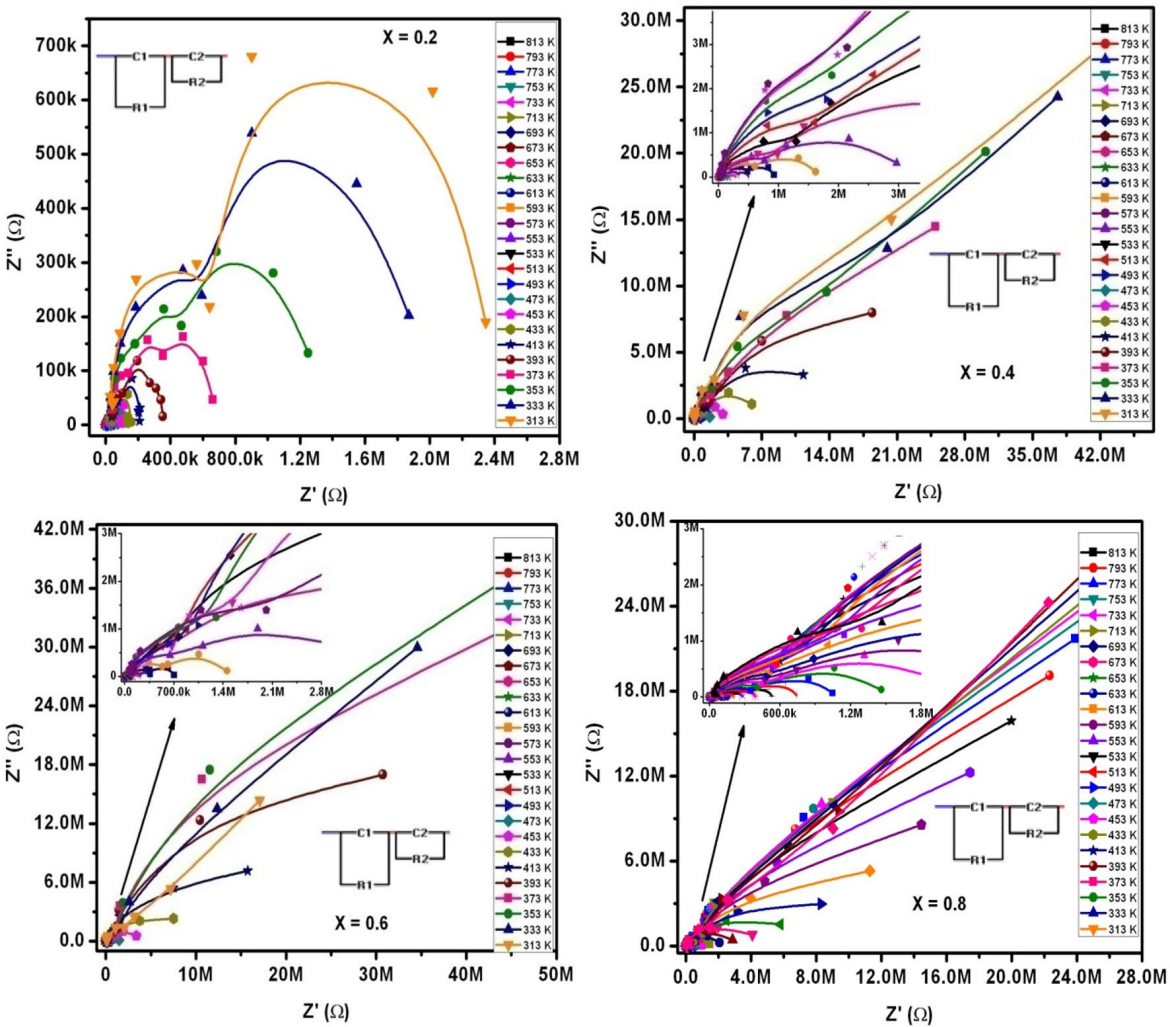
Z′ versus Z″ versus log f plots of SLFO nanoparticles.

As we know that the complex impedance (Z*) parameter can provide the information about the behavior of microstructure of polycrystalline materials. Further to elucidate the electrical conduction mechanism of as prepared SLFO-nanoparticles. The complex impedance relation of Z* = Z′−jZ″, where Z′ & Z″ are real and imaginary parts of complex impedance parameter can be useful to discuss impedance spectroscopy. Besides, the Cole–Cole plots (Nyquist plots) were plotted as displayed in Fig. 19. Nyquist plots were used to describe the conduction mechanism as a function of increase of La-concentration in SLFO samples at various temperatures ranging from 313 to 813 K. Clearly, two semicircular arcs were found for X = 0.2–0.8 contents. In particular, X = 0.2 composition exhibited two semicircles, whereas the remaining compositions showed slightly weak arcs. The partial relaxation strength may be accountable for the presence of weak arcs^25^. Similarly, the ions moving for longer distances can induce the formation of partial arcs. The X = 0.2 content attained complete relaxation strength owing to the existence of few ions confining to potential well^25^. The existence of semicircles indicated the magnetic semiconducting behavior for X = 0.2 samples. In case of two arcs, the first arc indicates the nature of grain whereas second one connected to grain-boundary. Normally, grain is high conductive layer and its boundary acts as low conductive layer. In current work, the Nyquist-plots were thoroughly examined via Z-view software (utilizing two RC-circuits). Accordingly, the accomplished values of grain/grain-boundary resistance (R_g_(R_1_)/R_gb_(R_2_)) with their respective capacitances ((C_g_(C_1_)/C_gb_(C_2_))). The obtained values were tabulated in Tables 4, 5, 6 and 7. This was evident that for all ‘X’ values, the values of grain-resistance and grain-boundary resistance were observed to be decreasing with increase of temperature (from 313 to 813 K). Consequently, the corresponding capacitance values (C_g_ & C_gb_) were noticed to be increasing with temperature. Comparatively, the grain boundaries exhibit large resistance than the grains. Using the intersecting portion of first and second arcs at Z′-axis, the parameters like R_g_ and R_gb_ were elucidated. Therefore, first arc delivered R_g_, whereas second one showed R_gb_. This established a fact the grain boundaries consist of few conductive layers while the grains constitute more conductive layers. Practically this was demonstrated from bulk conductivity of grain (σ_g_ = t/R_g_A, where ‘t’ = thickness, and ‘A’ = area of cross section of pellet) and its boundary (σ_gb_ = t/R_gb_A) (See Tables 4, 5, 6, 7). The obtained results confirmed that grains accomplished larger electrical conductivity than grain boundaries. Hence, it was clear that the current results satisfied the Koop’s theory^36^. In addition, the electrical conductivity of X = 0.2–0.8 contents were found to be increasing with temperature and hence, it obeyed the Arrhenius law as mentioned in the previous work^25^. Apart from this, the induced relaxations were also observed. For this, we assumed the centers of arcs and noticed that if the centers of arcs were placed below real axis. Therefore, it can be recommended that the non-Debye relaxations were observed for x = 0.2–0.8^25^. In this view, the relaxation time constants (τ) of grains (τ_g_) and grain-boundaries (τ_gb_) were computed. The obtained results (see Tables 4, 5, 6, 7) ensured that the time constant was increased with increase of ‘T’. Moreover, the second arcs expressed high ‘τ_gb_’ values.

**Table 4:** Impedance spectroscopy parameters of X = 0.2 content.

T (K)	R_g_ (Ω)	R_gb_ (Ω)	C_g_ (F)	C_gb_ (F)	σ_g_ (S/cm)	σ_gb_ (S/cm)	τ_g_ (s)	τ_gb_ (s)
**X = 0.2**								
313	799,444	2,446,922	8.01E−14	4.71E−12	8.83E−09	2.89E−09	6.40E−08	1.15E−05
333	774,961	1,995,912	2.87E−13	6.37E−12	9.11E−09	3.54E−09	2.22E−07	1.27E−05
353	636,436	1,406,376	4.36E−13	8.21E−12	1.11E−08	5.02E−09	2.77E−07	1.15E−05
373	444,434	697,000	6.48E−13	2.57E−11	1.59E−08	1.01E−08	2.88E−07	1.79E−05
393	173,536	379,385	9.23E−13	4.49E−11	4.07E−08	1.86E−08	1.60E−07	1.70E−05
413	148,109	230,729	9.88E−13	6.75E−11	4.77E−08	3.06E−08	1.46E−07	1.56E−05
433	62,115	167,080	2.94E−12	7.11E−11	1.14E−07	4.23E−08	1.83E−07	1.19E−05
453	53,804	125,336	3.15E−12	9.03E−11	1.31E−07	5.63E−08	1.69E−07	1.13E−05
473	49,663	102,425	3.75E−12	9.78E−11	1.42E−07	6.89E−08	1.86E−07	1.00E−05
493	41,322	81,456	4.62E−12	2.55E−10	1.71E−07	8.67E−08	1.91E−07	2.08E−05
513	35,518	63,156	5.11E−12	3.74E−10	1.99E−07	1.12E−07	1.81E−07	2.36E−05
533	27,444	49,580	6.64E−12	5.06E−10	2.57E−07	1.42E−07	1.82E−07	2.51E−05
553	21,701	38,004	7.32E−12	5.99E−10	3.25E−07	1.86E−07	1.59E−07	2.28E−05
573	17,360	27,571	8.05E−12	6.48E−10	4.07E−07	2.56E−07	1.40E−07	1.79E−05
593	12,580	20,888	9.68E−12	7.44E−10	5.61E−07	3.38E−07	1.22E−07	1.55E−05
613	9004	16,082	1.56E−11	8.52E−10	7.84E−07	4.39E−07	1.40E−07	1.37E−05
633	6832	13,662	3.12E−11	9.09E−10	1.03E−06	5.17E−07	2.13E−07	1.24E−05
653	4714	10,479	4.72E−11	9.84E−10	1.5E−06	6.74E−07	2.23E−07	1.03E−05
673	2321	8150	5.35E−11	4.94E−09	3.04E−06	8.67E−07	1.24E−07	4.03E−05
693	1489	6220	6.25E−11	6.16E−09	4.74E−06	1.14E−06	9.31E−08	3.83E−05
713	1054	5011	7.15E−11	9.41E−09	6.7E−06	1.41E−06	7.54E−08	4.72E−05
733	836	3935	2.12E−10	3.68E−08	8.45E−06	1.79E−06	1.77E−07	1.45E−04
753	528	3177	9.36E−10	4.57E−08	1.34E−05	2.22E−06	4.94E−07	1.45E−04
773	317	2355	2.48E−09	5.82E−08	2.23E−05	3E−06	7.86E−07	1.37E−04
793	280	1774	4.42E−09	6.97E−08	2.52E−05	3.98E−06	1.24E−06	1.24E−04
813	195	1646	6.08E−09	8.60E−08	3.62E−05	4.29E−06	1.19E−06	1.42E−04

**Table 5:** Impedance spectroscopy parameters of X = 0.4 content.

T (K)	R_g_ (Ω)	R_gb_ (Ω)	C_g_ (F)	C_gb_ (F)	σ_g_ (S/cm)	σ_gb_ (S/cm)	τ_g_ (s)	τ_gb_ (s)
**X = 0.4**								
313	4,433,887	4.9E+07	5.30E−14	2.58E−12	1.59E−09	1.44E−10	2.35E−07	1.26E−04
333	3,841,257	4.2E+07	8.97E−14	3.29E−12	1.84E−09	1.7E−10	3.45E−07	1.37E−04
353	3,281,653	3.4E+07	1.43E−13	4.07E−12	2.15E−09	2.08E−10	4.69E−07	1.38E−04
373	2,977,422	3.2E+07	3.33E−13	7.69E−12	2.37E−09	2.22E−10	9.91E−07	2.44E−04
393	2,766,950	2.9E+07	4.68E−13	2.49E−11	2.55E−09	2.4E−10	1.29E−06	7.32E−04
413	2,588,862	1.6E+07	4.99E−13	2.45E−11	2.73E−09	4.41E−10	1.29E−06	3.92E−04
433	2,264,920	7,754,721	9.26E−13	3.60E−11	3.12E−09	9.11E−10	2.10E−06	2.79E−04
453	1,944,513	3,937,347	9.96E−13	4.55E−11	3.63E−09	1.79E−09	1.94E−06	1.79E−04
473	753,442	2,059,000	2.17E−12	3.91E−11	9.37E−09	3.43E−09	1.63E−06	8.05E−05
493	494,478	1,327,717	1.54E−12	7.60E−11	1.43E−08	5.32E−09	7.61E−07	1.01E−04
513	308,157	791,443	2.75E−12	1.17E−10	2.29E−08	8.92E−09	8.47E−07	9.26E−05
533	236,740	306,541	3.40E−12	2.73E−10	2.98E−08	2.3E−08	8.05E−07	8.37E−05
553	150,018	194,131	3.69E−12	3.13E−10	4.71E−08	3.64E−08	5.54E−07	6.08E−05
573	92,730	127,932	4.00E−12	3.33E−10	7.62E−08	5.52E−08	3.71E−07	4.26E−05
593	71,552	98,808	4.70E−12	3.74E−10	9.87E−08	7.15E−08	3.36E−07	3.70E−05
613	73,100	84,360	7.65E−12	4.20E−10	9.66E−08	8.37E−08	5.59E−07	3.54E−05
633	60,046	62,790	9.03E−12	4.79E−10	1.18E−07	1.12E−07	5.42E−07	3.01E−05
653	40,167	56,235	2.58E−11	3.98E−10	1.76E−07	1.26E−07	1.04E−06	2.24E−05
673	18,634	22,902	2.85E−11	1.68E−09	3.79E−07	3.08E−07	5.31E−07	3.85E−05
693	10,176	13,084	3.24E−11	3.20E−09	6.94E−07	5.4E−07	3.30E−07	4.19E−05
713	6442	9950	3.62E−11	4.92E−09	1.1E−06	7.1E−07	2.33E−07	4.90E−05
733	4094	8152	5.77E−11	1.14E−08	1.72E−06	8.66E−07	2.36E−07	9.29E−05
753	2729	5631	4.56E−10	1.52E−08	2.59E−06	1.25E−06	1.24E−06	8.56E−05
773	1640	4543	7.30E−10	3.05E−08	4.31E−06	1.55E−06	1.20E−06	1.39E−04
793	1080	3258	2.46E−09	2.54E−08	6.54E−06	2.17E−06	2.66E−06	8.28E−05
813	203	2812	3.16E−09	3.24E−08	3.48E−05	2.51E−06	6.41E−07	9.11E−05

**Table 6:** Impedance spectroscopy parameters of X = 0.6 content.

T (K)	R_g_ (Ω)	R_gb_ (Ω)	C_g_ (F)	C_gb_ (F)	σ_g_ (S/cm)	σ_gb_ (S/cm)	τ_g_ (s)	τ_gb_ (s)
**X = 0.6**								
313	2,809,385	2.7E+07	4.27E−14	2.93E−12	2.51E−09	2.59E−10	1.20E−07	8.00E−05
333	2,571,146	7.4E+07	8.75E−14	3.80E−13	2.75E−09	9.56E−11	2.25E−07	2.81E−05
353	2,462,010	7.5E+07	7.50E−14	2.76E−13	2.87E−09	9.43E−11	1.85E−07	2.07E−05
373	2,336,000	7E+07	1.86E−13	6.19E−12	3.02E−09	1.01E−10	4.34E−07	4.32E−04
393	2,205,940	5.6E+07	3.50E−13	2.82E−11	3.2E−09	1.26E−10	7.72E−07	1.59E−03
413	2,008,327	3.8E+07	3.88E−13	4.00E−11	3.52E−09	1.84E−10	7.79E−07	1.54E−03
433	1,642,555	9,442,031	4.10E−13	4.19E−11	4.3E−09	7.48E−10	6.73E−07	3.96E−04
453	989,936	4,826,410	4.20E−13	5.34E−11	7.13E−09	1.46E−09	4.16E−07	2.58E−04
473	681,423	974,621	1.43E−12	5.79E−11	1.04E−08	7.25E−09	9.74E−07	5.64E−05
493	449,065	638,415	8.90E−13	9.08E−11	1.57E−08	1.11E−08	4.00E−07	5.80E−05
513	322,459	444,472	1.14E−12	2.43E−10	2.19E−08	1.59E−08	3.68E−07	1.08E−04
533	106,224	195,410	1.94E−12	2.12E−10	6.65E−08	3.61E−08	2.06E−07	4.14E−05
553	52,809	72,166	2.30E−12	3.60E−10	1.34E−07	9.79E−08	1.21E−07	2.60E−05
573	31,140	55,004	2.68E−12	3.86E−10	2.27E−07	1.28E−07	8.35E−08	2.12E−05
593	27,468	49,372	3.53E−12	3.36E−10	2.57E−07	1.43E−07	9.70E−08	1.66E−05
613	19,662	32,208	7.13E−12	4.92E−10	3.59E−07	2.19E−07	1.40E−07	1.58E−05
633	10,061	16,847	1.10E−12	5.64E−10	7.02E−07	4.19E−07	1.11E−08	9.50E−06
653	7328	11,425	9.40E−12	5.87E−10	9.64E−07	6.18E−07	6.89E−08	6.71E−06
673	6618	9182	1.27E−11	3.05E−09	1.07E−06	7.69E−07	8.40E−08	2.80E−05
693	4299	8704	1.74E−11	2.69E−09	1.64E−06	8.11E−07	7.48E−08	2.34E−05
713	2049	6722	2.21E−11	5.80E−09	3.45E−06	1.05E−06	4.53E−08	3.90E−05
733	1688	5811	4.84E−11	2.40E−08	4.18E−06	1.22E−06	8.17E−08	1.39E−04
753	1007	4049	3.36E−10	1.86E−08	7.01E−06	1.74E−06	3.38E−07	7.53E−05
773	628	2965	6.72E−10	3.51E−08	1.12E−05	2.38E−06	4.22E−07	1.04E−04
793	377	2081	7.80E−10	4.11E−08	1.87E−05	3.39E−06	2.94E−07	8.55E−05
813	160	1832	1.65E−09	4.96E−08	4.41E−05	3.85E−06	2.64E−07	9.09E−05

**Table 7:** Impedance spectroscopy parameters of X = 0.8 content.

T (K)	R_g_ (Ω)	R_gb_ (Ω)	C_g_ (F)	C_gb_ (F)	σ_g_ (S/cm)	σ_gb_ (S/cm)	τ_g_ (s)	τ_gb_ (s)
**X = 0.8**								
313	916,647	3.6E+07	6.89E−14	2.53E−12	7.70E−09	1.94E−10	6.32E−08	9.23E−05
333	730,846	3.4E+07	1.28E−13	3.66E−12	9.66E−09	2.09E−10	9.35E−08	1.24E−04
353	583,249	2.2E+07	2.29E−13	4.92E−12	1.21E−08	3.27E−10	1.34E−07	1.06E−04
373	394,307	1.4E+07	3.74E−13	1.07E−11	1.79E−08	5.09E−10	1.47E−07	1.49E−04
393	208,432	8,664,420	5.89E−13	2.38E−11	3.39E−08	8.15E−10	1.23E−07	2.06E−04
413	123,684	7,946,210	6.38E−13	3.92E−11	5.71E−08	8.89E−10	7.89E−08	3.11E−04
433	84,267	5,318,240	3.20E−13	4.17E−11	8.38E−08	1.33E−09	2.70E−08	2.22E−04
453	62,358	5,006,218	1.47E−12	5.68E−11	1.13E−07	1.41E−09	9.17E−08	2.84E−04
473	42,367	3,649,201	1.88E−12	6.26E−11	1.67E−07	1.94E−09	7.96E−08	2.28E−04
493	29,498	3,000,872	2.47E−12	2.40E−11	2.39E−07	2.35E−09	7.29E−08	7.20E−05
513	15,360	2,585,484	2.80E−12	1.87E−10	4.6E−07	2.73E−09	4.30E−08	4.83E−04
533	12,189	1,509,849	3.85E−12	2.77E−10	5.79E−07	4.68E−09	4.69E−08	4.18E−04
553	10,364	724,501	4.31E−12	3.40E−10	6.81E−07	9.75E−09	4.47E−08	2.46E−04
573	8476	583,166	4.81E−12	3.74E−10	8.33E−07	1.21E−08	4.08E−08	2.18E−04
593	9410	302,206	5.92E−12	4.39E−10	7.5E−07	2.34E−08	5.57E−08	1.33E−04
613	10,868	188,804	2.60E−12	5.13E−10	6.5E−07	3.74E−08	2.83E−08	9.69E−05
633	8087	72,819	1.45E−11	6.06E−10	8.73E−07	9.7E−08	1.17E−07	4.41E−05
653	6410	56,220	2.54E−11	6.37E−10	1.1E−06	1.26E−07	1.63E−07	3.58E−05
673	5503	38,462	2.97E−11	2.69E−09	1.28E−06	1.84E−07	1.63E−07	1.03E−04
693	3038	10,999	3.58E−11	3.52E−09	2.32E−06	6.42E−07	1.09E−07	3.87E−05
713	1118	7544	4.19E−11	6.28E−09	6.32E−06	9.36E−07	4.68E−08	4.74E−05
733	628	5008	7.64E−11	1.83E−08	1.12E−05	1.41E−06	4.80E−08	9.16E−05
753	447	4892	5.70E−10	2.43E−08	1.58E−05	1.44E−06	2.55E−07	1.19E−04
773	308	3529	1.01E−09	3.29E−08	2.29E−05	2E−06	3.11E−07	1.16E−04
793	364	2758	2.33E−09	4.07E−08	1.94E−05	2.56E−06	8.48E−07	1.12E−04
813	149	2420	3.46E−09	5.18E−08	4.74E−05	2.92E−06	5.16E−07	1.25E−04

## Conclusions

In this study, the SLFO nanoparticles were prepared using the hydrothermal method varying the La-content from X = 0.2–0.8. The diffraction pattern evidenced the hexagonal crystal structure. In addition, the lattice constants were observed to be increasing from 0.58801 to 0.58825 nm (a = b), and 2.30309 to 2.30341 nm (c) with increase in ‘X’. The FESEM and TEM pictures indicated the spheres like grains as well as particles in the morphology. Furthermore, the E_g_ values were identified to be increasing from 1.866 to 2.118 eV with increase in ‘X’. The M–H loops of SLFO revealed the decreasing trend of magnetization from 36.34 to 7.17 emu/g with increase in ‘X’. The X = 0.6 content revealed the high dielectric constant (~ 18) and dielectric loss (10.85) at high frequencies. Therefore, this composition can be suitable for dielectric absorber applications at 1–5 MHz. The E_a_ values of H-region were altered from 0.147 to 0.190 eV while the L-region showed the same trend changing from 0.0274 to 0.0443 eV. Using the power law fit, the dc-conductivity and exponents were computed. The dielectric modulus formalism provided the clear evidence for the space charge polarization mechanism. Besides, the short range and long-range hopping conduction mechanism was described. The Cole–Cole plots showed the elucidation of grain and grain boundary contribution in the electrical conduction mechanism. The obtained results indicated that the grains accomplished more electrical conductivity than the grain-boundaries.

## Data Availability

Date will not be provided publicly by all authors, and it will be provided immediately based on suitable request to the corresponding author.
